# Introgression line libraries in melon (*Cucumis melo* L.): development, genetic insights, and applications in trait dissection and crop improvement

**DOI:** 10.1007/s11032-026-01725-x

**Published:** 2026-10-06

**Authors:** Cristina Esteras, Marta Pujol, Juan Pablo Fernández-Trujillo, Maria José Gonzalo, Ana Monserrat Martín-Hernández, Ana María Pérez-de-Castro, Belén Picó, Jordi Garcia-Mas, Antonio José Monforte

**Affiliations:** 1 Instituto de Conservación y Mejora de La Agrodiversidad Valenciana (COMAV-UPV), Universitat Politècnica de València, Camino de Vera S/N, Valencia, 46022 Spain; 2 Centre for Research in Agricultural Genomics (CRAG), CSIC-IRTA-UAB-UB, Edifici CRAG, Campus UAB, Bellaterra, Barcelona, 08193 Spain; 3 Institut de Recerca I Tecnología Agroalimentàries (IRTA), Edifici CRAG, Campus UAB, Bellaterra, Barcelona, 08193 Spain; 4 Department of Agronomical Engineering, Technical University of Cartagena (UPCT), Paseo Alfonso XIII, 48, ETSIA, Cartagena, Murcia 30203 Spain; 5 Instituto de Biología Molecular y Celular de Plantas (IBMCP), Consejo Superior de Investigaciones Científicas (CSIC), Universitat Politècnica de València (UPV), Ingeniero Fausto Elio S/N, Valencia, 46022 Spain

**Keywords:** QTL, Fruit morphology, Ripening, Germplasm, Cloning, Breeding, Markers assisted selection

## Abstract

Introgression lines (ILs) are powerful genetic resources for dissecting the architecture of complex traits in crops. This review summarizes the development and characterization of six IL libraries and two additional ILs in melon (*Cucumis melo* L.), generated from diverse donor genotypes, including wild accessions, landraces, traditional and elite cultivars, introgressed into Mediterranean elite backgrounds, such as ‘Piel de Sapo’ and ‘Védrantais.’ Together, these libraries capture a wide representation of melon genetic diversity and have revealed numerous stable quantitative trait loci (QTLs) for key agronomic traits, including fruit shape, weight, color, firmness, soluble solids content, volatile composition, climacteric ripening, and resistance to Cucumber Mosaic Virus (CMV). Notably, large-effect QTLs were recurrently identified across independent IL libraries. This suggests that targeted improvement of complex traits may be achieved by manipulating a limited number of loci. Notably, several ILs displayed transgressive phenotypes relative to their donor parents. These results highlight their ability to uncover cryptic genetic variants relevant to modern breeding. Beyond QTL discovery, ILs have facilitated fine mapping and gene identification, leading to the cloning of genes such as *CmOFP13* (fruit shape), *CmNAC-NOR*, *CmERF024* (ripening), and *CmVPS41* (CMV resistance). Overall, this review highlights the value of ILs as tools for both breeding and fundamental genetic research and outlines future directions, including integrating transcriptomics and metabolomics approaches, developing ILs for abiotic stress tolerance, and extending IL resources to oriental melon germplasm, thereby enhancing their utility for global melon improvement.

**Supplementary Information:**

The online version contains supplementary material available at https://doi.org/10.1007/s11032-026-01725-x.

## Key message

This review demonstrates that multi-donor melon introgression line (IL) libraries bridging wild, landrace, and elite germplasm provide a powerful framework to resolve the polygenic architecture of complex agronomic traits, enabling the detection of stable and large-effect QTLs, the identification of causal genes, and the translation of genetic diversity into targeted breeding strategies for fruit quality, ripening, and disease resistance.

**Supplementary Information:**

The online version contains supplementary material available at https://doi.org/10.1007/s11032-026-01725-x.

## Introduction

Melon (*Cucumis melo* L.) is a horticultural crop of notable economic importance, widely cultivated across a broad range of agroclimatic zones. As reported by FAOSTAT (https://www.fao.org/faostat/es/#data/QCL, accessed 29 June 2026), global production has reached approximately 28 million tons per year, reflecting a 9% increase over the past decade, despite a modest 2% decline in harvested area. This improvement in yield efficiency (an 11% kg/ha increase) is likely driven by recent advances in breeding strategies, particularly those targeting resistance to biotic stresses, alongside the adoption of more efficient agronomic practices.

The melon species belongs to the *Cucurbitaceae* family. This family contains about 1000 species and 96 genera (Renner and Schaefer 2017). *Cucurbitaceae* originated in Southeast Asia and spread worldwide. Consequently, several cucurbit species have been domesticated historically in different continents and nowadays they are widely valued crops: melon (Asia and Africa), cucumber (*C. sativus* L., Asia) watermelon (*Citrullus lanatus* (Thunb.) Matsum. & Nakai, Africa), pumpkins and squashes (*Cucurbita pepo* L., *C. moschata* Duchesne ex Poir, *C. maxima* Duchesne ex Lam., *C. argyrosperma* Huber, *C. ficifolia* Bouché*,* America), chayote (*Sechium edule* (Jacq.) Sw., America), gourds (*Momordica charantia* L., *Lagenaria siceraria* (Molina) Standl., *Benincasa hispida* (Thunb.) Cogn., *Luffa aegyptiaca* Mill., *Luffa acutangula* (L.) Roxb., *Trichosanthes cucumerina* L., Africa and Asia), among other minor crops. Overall, cucurbit crops share two characteristics: they were independently domesticated on every continent where they were present, and they are capable of producing exceptionally large fruits, reaching up to 1 ton in some *C. maxima* cultivars.

*C. melo* originated in Asia/Australia (Sebastian et al. 2010) and was domesticated independently in Central Asia and East Africa (Zhao et al. 2019). Consequently, two subspecies have been defined (Endl et al. 2018): subsp. *meloides* (African origin) and subsp. *melo* (Asian origin). Cultivars belonging to subsp. *meloides* are currently found in the Sudan region. In contrast, subsp. *melo* spread from its geographical origin in the Indian subcontinent (the primary center of diversity) both eastward and westward in a divergent manner, with each diversification including different gene pools (Gonzalo et al. 2019; Zhao et al. 2019). Genetic diversity in melon follows a marked geographic gradient, with Indian germplasm representing the center of diversity and Mediterranean and East Asian cultivars occupying opposite extremes of the distribution (Gonzalo et al. 2019). Consequently, Mediterranean and East Asian melons are among the most genetically differentiated cultivated groups within the species. This large genetic divergence explains the value of East Asian germplasm for trait discovery in Mediterranean breeding backgrounds and vice versa. The genetic diversity shows clear geographical cline, with cultivars from India in the center of the distribution and Mediterranean and East Asian cultivars in the extremes of the distribution.

Originally, melon cultivars were not sweet, their culinary uses were as vegetables: raw, cooked, or pickled. Non-sweet melons arrived in the Mediterranean Basin during Ancient Egyptian times (Janick et al. 2007). Sweet melons emerged in the ninth century CE in central Asia and were introduced in Europe between the tenth and fifteenth centuries (Paris et al. 2012). Sweet melons are the most popular and have a higher market value nowadays, but non-sweet melons are still cultivated and are highly appreciated in local markets in Asian, Mediterranean, and North African countries, such as the snake melons Fakous in the Near-East (Merheb et al. 2020), Alficoz in Spain (Flores-León et al. 2021), and Carosello cultivars in Italy (Pavan et al. 2017). As a consequence of this history of diversification, melon cultivars display a wide range of phenotypic diversity, especially in fruit characteristics, ranging from very small (30 g) to very large fruits (> 10 kg) with slightly flattened to highly elongated shapes, different rind (white to very dark green, yellow, red) and flesh (white, green, orange) colors, climacteric to non-climacteric ripening, wide variation in volatile composition, and low to high sugar content. In addition, a wide variation can be found in the tolerance and resistance to major diseases among traditional cultivars and landraces (Martín-Hernández and Picó 2021; Shahwar et al. 2024). Currently, traditional and modern cultivars display different combinations of these characteristics. Pitrat (2017) proposed a comprehensive horticultural classification in 19 groups: agrestis, kachri, chito, tibish, acidulus, momordica, conomon, makuwa, chinensis, flexuosus, chate, dudaim, chandalak, indicus, ameri, cassaba, ibericus, inodorus, and cantalupensis.

The genetic basis of phenotypic diversity in melon germplasm has been investigated in numerous quantitative trait locus (QTL) mapping studies. A search on the Web of Science (https://www.webofscience.com/wos/woscc/basic-search, 1 September 2026) with the keywords “melon and QTL” retrieved 220 articles. Most research has focused on fruit quality traits and disease resistance (Powdery and Downy mildew, Fusarium, and different viruses) and, to a lesser extent, on seed and vegetative traits. These researches have been reviewed in several scientific articles (Díaz et al. 2011; Monforte et al. 2014; Gonzalo and Monforte 2017; Pan et al. 2020; Martín-Hernández and Picó 2021; Pujol and Garcia-Mas 2023; Shahwar et al. 2024; Mayobre et al. 2024a; Tzuri et al. 2025). These studies have revealed the complex genetic architecture of agronomic traits in melon and have laid the groundwork for gene identification (Feder et al. 2015; Rios et al. 2017; Guiu-Aragonés et al. 2015; Martinez-Martinez et al. 2022; Wang et al. 2022; Tang et al. 2025).

QTL mapping in melon has been carried out in synthetic populations as Recombinant Inbred Lines (RIL: Périn et al. 2002a; Zalapa et al. 2007; Yuste-Lisbona et al. 2011; Sherman et al. 2013; Pereira et al. 2018; Branham et al. 2018; Pérez-de-Castro et al. 2019), F_2_ populations (Monforte et al. 2004; Park et al. 2009; Fukino et al. 2012; Amanullah et al. 2021; Cao et al. 2021; Lian et al. 2021), and to a lesser extent Double Haploid Lines (DHL, Monforte et al. 2004) and Genome-Wide Association Analysis (GWAS, Tomason et al. 2013; Gur et al. 2017), and Introgression Lines (IL, Campos et al. 2023; Castro et al. 2019; Díaz et al. 2014; Eduardo et al. 2007; Pereira et al. 2021; Santo Domingo et al. 2022a).

Among the different mapping populations, this review focuses on the development and study of ILs in melon genetic research.

An IL is an inbred line that contains a single or very few introgressions from a donor genotype into a recipient variety. Therefore, the IL is Near-Isogenic to the recipient variety; therefore, it will be very similar phenotypically to it, except for the possible effect of the alleles from the donor genotype located in the introgressed chromosome. Introgression lines have been used extensively in classical breeding, for example, to introduce disease resistance genes from wild species into modern cultivars. Eshed and Zamir (1994) expanded the concept developing a set of IL from the wild tomato *Solanum pennellii* accession LA 716 in the processing tomato cultivar M82, each IL containing a single introgression, and the introgressions represented the genome of LA 716. The collection of ILs was defined as a genomic library of the wild species in the cultivated background. This IL library has been used extensively in tomato research as: QTL cloning (Fridman et al. 2000), deep characterization of yield QTLs (Gur and Zamir 2004; Semel et al. 2006; Gur et al. 2010), changes in gene expression (Baxter et al. 2005), fruit volatile composition and flavor (Tieman et al. 2006; Liu et al. 2016; Kaur et al. 2023). Additional IL libraries have been developed in tomato using different wild species as *S. pimpinellifolium* (Barrantes et al. 2014), *S. habrochaites* (Monforte and Tanksley 2000; Finkers et al. 2007), *S. chmielewskii* (Prudent et al. 2009), *Solanum neorickii* (Brog et al. 2019), *S. lycopersicoides* (Canady et al. 2005), *S. sitiens* (Chetelat et al. 2019).

IL libraries have been also developed in other species such as *Arabidopsis thaliana* (Lisec et al. 2008), barley (von Korff et al. 2004; Schmalenbach et al. 2009; Honsdorf et al. 2017), cabbage (Ramsay et al. 1996), eggplant (Gramazio et al. 2017), lettuce (Jeuken and Lindhout 2004), peach (Kalluri et al. 2022), pearl millet (Kumari et al. 2014), strawberry (Urrutia et al. 2016), and wheat (King et al. 2022). A special mention should be made of rice, where thousands of ILs are available, detecting thousands of QTLs, identifying 31 genes involved in several relevant agronomic traits, and incorporating ILs into breeding programs (Zhang et al. 2022a).

All the previous works demonstrated the advantages of using IL libraries to dissect genetically complex traits: i) “domestication” of exotic genetic variability, ii) estimation of single QTL effects on the appropriate genetic background (GB), iii) study at single QTL level complex genetic interactions as QTL-x-Environment, QTL-x-GB, iv) study of epistatic interaction among QTLs, v) controlled combination of multiple QTLs in a single breeding line, and vi) QTL cloning.

Introgression line development have also been reported in cucurbit crops. In cucumber, the wild relative *Cucumis hystrix* has been used as a donor to generate interspecific IL populations (Li et al. 2020). Subsequent studies have exploited individual ILs to investigate specific traits, including fruit and ovary morphology (Wang et al. 2023, 2024), resistance to *Aphis gossypii* (Ning et al. 2025), *Meloidogyne incognita* (Wang et al. 2020), and powdery mildew (Zhang et al. 2018). Most of these studies have been based on genetic materials derived from the *C. hystrix* introgression platform. In watermelon, only early-stage introgression populations have been reported to date (Al-Qadumii et al. 2025). Likewise, very few examples of IL development have been described in *Cucurbita* spp. (e.g. Sun et al. 2026). In contrast, melon possesses multiple donor-derived IL libraries representing highly divergent genetic backgrounds and horticultural groups. Consequently, melon currently constitutes one of the most extensively developed and broadly exploited introgression-line systems within the *Cucurbitaceae*.

The first melon IL library was constructed by Eduardo et al. (2005) using the Korean accession PI 161375 cv. ‘Songwhan Charmi’ (SC) as donor genotype and an elite Spanish Piel de Sapo cultivar (PS-T111). In order to obtain a broader representation of melon germplasm, five additional IL libraries and single ILs were built thereafter with donor genotypes from different world regions, in a collaborative effort among Spanish research groups belonging to IRTA-CRAG, UPV and IBMCP-CSIC-UPV (Campos et al. 2023; Castro et al. 2019; Díaz et al. 2014; Pereira et al. 2021; Perpiñá et al. 2016; Santo Domingo et al. 2022a; Supplementary Files 1 and 2). These libraries have been studied extensively compiling a thorough knowledge on the genetic control of relevant agronomic traits (Supplementary File Cite ESM.1). In this review, we will show the major achievements obtained from this research and we will discuss possible future objectives related to melon introgression lines.

## Melon introgression lines

Wild accessions are typically used as donors for IL libraries because they harbor genetic variation not present within the cultivated gene pool, making them valuable sources of novel alleles for breeding (Zamir 2001). However, interspecific crosses between *C. melo* and wild *Cucumis* species are not feasible because of reproductive barriers that prevent the formation of fertile hybrids (Ferriol et al. 2023). Nevertheless, the genetic diversity within *C. melo* is sufficiently broad to support the implementation of introgression line strategies. This diversity includes wild *C. melo* accessions and highly differentiated cultivars from geographically distant regions, whose gene pools can be considered nearly as exotic as those of wild species (Gonzalo et al. 2019).

**Fig. 1:**
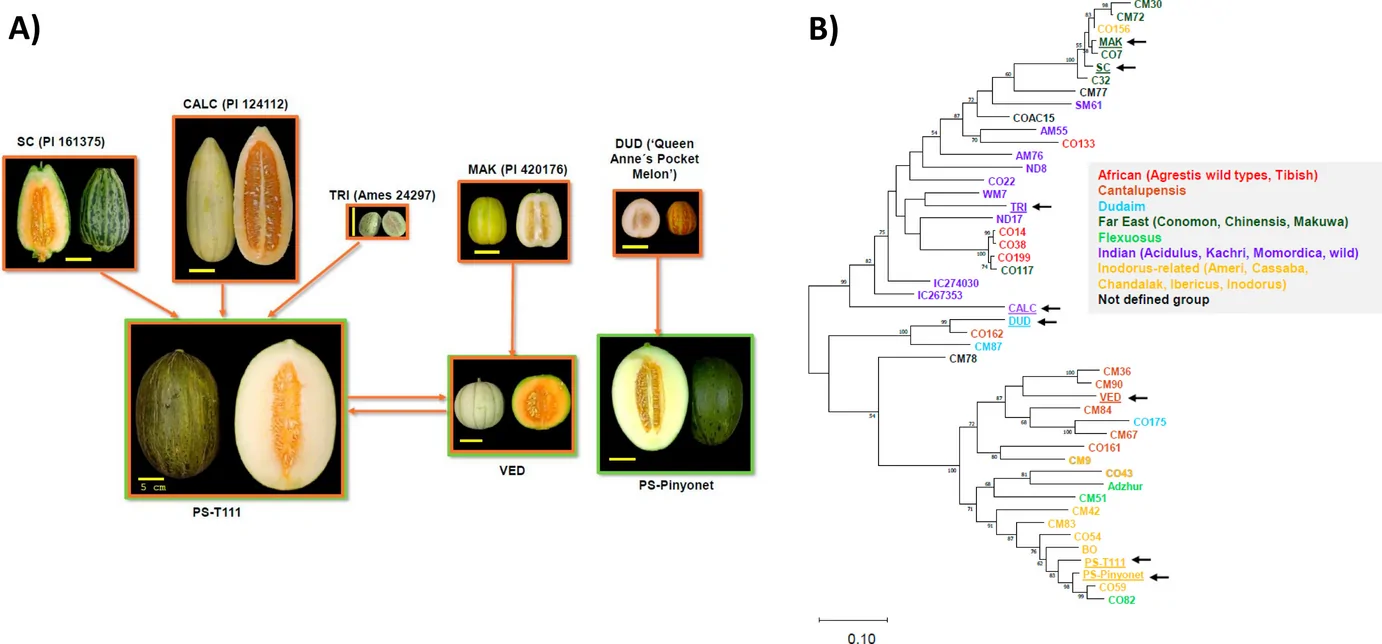
Accessions selected for the development of the introgression lines (ILs). **A** Representative fruit images of the parental accessions: PI 161375 (SC), PI 124112 (CALC), Ames 24297 (TRI), PI 420176 (MAK), ‘Queen Anne’s Pocket Melon’ (DUD), ‘Piel de Sapo’ (PS-T111) and ‘Pinyonet’ (PS-Pinyonet), and ‘Védrantais’ (VED). The yellow bar indicates the scale (5 cm). Green and orange frames denote the recurrent (recipient) and donor genotypes, respectively. Crosses are indicated by arrows. **B** Diversity and phylogenetic relationships of the IL parents relative to a worldwide panel of representative melon accessions based on several thousand filtered SNPs from the GBS dataset of 175 accessions published by Gonzalo et al. (2019). The maximum Likelihood phylogenetic tree (Nei and Kumar 2000) was generated using MEGA v 12.1 (Kumar et al. 2024) with a filtered subset of 48 accessions and 1,617 SNPs. The tree with the highest log likelihood (–30,678.32) is shown here. Bootstrap values (1,000 replicates; Felsenstein 1985) are displayed at the nodes. Branch lengths represent the number of substitutions per site, with a scale bar shown below the tree. Parental lines of the ILs are underlined and marked with arrows. Colors represent melon groups following Pitrat (2017), simplified according to Gonzalo et al. (2019)

The selected recurrent genotypes represented two of the most popular market classes in Europe: ‘Piel de Sapo’ (PS) and Cantaloupe. The ‘Piel de Sapo’ market class belongs to the ibericus group (Pitrat 2017), cultivated mostly in the Iberian Peninsula, but also in North and South America. Two PS inbred lines were selected as background genotypes: PS-T111, an elite inbred from Semillas Fitó S.A. (Eduardo et al. 2005; Díaz et al. 2014; Santo Domingo et al. 2022a; Campos et al. 2023), and ‘Pinyonet’ (PS-Pinyonet), a traditional Spanish variety maintained by the Cucurbit Genetic Breeding group from the Genebank of the Institute for the Conservation and Breeding of Agrobiodiversity (COMAV-UPV) (Castro et al. 2019). The ‘Védrantais’ (VED), belonging to the charentais subgroup (Pitrat 2017), was selected as Cantaloupe background genotype. Charentais melons are the most widely cultivated type of the cantalupensis group in Europe, especially in France and the USA. ‘Piel de Sapo’ and charentais cultivars differ in many traits, such as fruit size and shape, rind features, aroma, flesh properties, sugar, organic acids and volatile composition, ripening features, and shelf life (Fig. 1A, Supplementary File 2).

Donor parents were selected to represent a broad spectrum of melon genetic diversity based on geographical origin genetic diversity analysis (Fig. 1B) and horticultural classification (Pitrat 2017). The distribution of the donor accessions across the melon diversity spectrum was intentionally designed to maximize allelic sampling and trait discovery potential. Indian accessions occupy the center of the species diversity distribution, whereas Mediterranean and Far Eastern cultivars are located at opposite ends of the diversity gradient (Gonzalo et al. 2019). Consequently, introgressions from Far Eastern and Indian germplasm into Mediterranean elite backgrounds are expected to introduce novel allelic combinations and uncover genetic variation absent from modern Mediterranean breeding pools. In addition, the inclusion of a wild agrestis accession (TRI) further expands the evolutionary range represented in the IL libraries and increases the probability of identifying loci associated with domestication-related traits. International accessions were kindly provided by the USDA National Plant Germplasm System (NPGS) collection. Additionally, reciprocal ILs were developed using PS-T111 and VED. Therefore, donor parents show high phenotypic diversity in terms of sex expression, fruit quality traits, and disease resistance (Fig. 1 A and Supplementary File 2).

The accession PI 161375, cultivar ‘Songwhan Charmi’ (SC), belongs to the chinensis group and clusters with other Far-East cultivar groups (Fig. 1B), being one of the accessions most genetically distant from ‘Piel de Sapo’ melons. The most remarkable fruit characteristics are low sugar content, pear shape, and green/orange flesh (Supplementary File 2). This accession is relevant for melon breeding and is one of the genotypes used in the first melon linkage maps (Périn et al. 2002a; Oliver et al. 2001; Gonzalo et al. 2005). Furthermore, the double haploid line (DHL) used in the assembly of the first reference melon genome (DHL92, Garcia-Mas et al. 2012) was obtained from a cross between SC and PS-T111. SC is resistant to several viruses (Martín-Hernández and Picó 2021; Dogimont and Sari 2022): the cucumovirus CMV (Cucumber mosaic virus, Essafi et al. 2009), the carmovirus MNSV (Melon necrotic spot virus, Coudriet et al. 1981), the tobamovirus KGMMV (Kyuri green mottle mosaic virus, Daryono et al. 2005), and the crinivirus BPYV (Beet pseudo-yellows virus, Esteva and Nuez 1992). In addition, SC shows partial resistance to the potyvirus Watermelon mosaic virus (WMV) and to the comovirus Squash mosaic virus (SqMV). Resistance to aphid-mediated virus transmission has also been reported in SC, as it carries the virus aphid transmission (*Vat)* gene (Dogimont et al. 2014), as well as resistance to soil-borne diseases such as *Fusarium oxysporum* f. sp. *melonis* (Risser 1973).

The Japanese cultivar ‘Ginsen Makuwa’, PI 420176 (MAK), belongs to the makuwa subgroup ginmakuwa*,* clustering with other Far-East groups (Fig. 1B). MAK produces sweet fruits with climacteric ripening behavior, intermediate between typically non-climacteric (such as PS) and climacteric (such as VED) melons, with a specific aroma profile and resistance to CMV (Pascual et al. 2019).

The accession PI 124112 (CALC) is an Indian landrace belonging to the momordica group, with an intermediate position in the phylogenetic tree (Fig. 1B between Far East and Mediterranean melons. The momordica group is quite heterogeneous and is mainly cultivated in India and Southeast Asia. CALC is relevant for melon breeding as it carries resistance genes to many viruses, such as the potyvirus PRSV (Papaya ringspot virus-watermelon type) (McCreight and Fashing-Burdette 1996), poliovirus CABYV (Cucumber aphid-borne yellows virus) (Dogimont et al. 1996), and the begomoviruses ToLCNDV (Tomato Leaf Curl New Delhi Virus) (López et al. 2017), CuLCrV (Cucurbit leaf crumple virus) (McCreight et al. 2008), and WmCSV (Watermelon chlorotic stunt virus) (Yousif et al. 2007); and also to fungal diseases such as powdery and downy mildew (Martín-Hernández and Picó 2021; Dogimont and Sari 2022).

The accession Ames 24,297 (TRI), originating from Pakistan, represents a typical wild melon classified in the agrestis group (Pitrat 2017). Phylogenetic analysis placed it in close association with Indian accessions (Fig. 1B). This accession produces non-edible fruits that are nearly fleshless and extremely small (< 50 g) (Fig. 1A, Supplementary File 2). The TRI IL library includes the only wild accession and is therefore particularly valuable for investigating domestication-related traits, such as fruit size and the increase in edible mesocarp.

The accession ‘Queen Anne’s Pocket Melon’ (CO180, DUD) was derived from PI 273438, originally collected in Iraq, and belongs to the dudaim group. This accession did not cluster with the Indian accessions (Fig. 1B). Dudaim melons are cultivated for ornamental purposes because of their pleasant fragrance, but they are not consumed. Their fruits are round and small, with a single yellow color and orange/ochre spots or stripes (Fig. 1A, Supplementary File 2). It was selected as a donor mainly because of its unique fragrance (Aubert and Pitrat 2006; Esteras et al. 2018, 2020). DUD is fully resistant to CMV (Pascual et al. 2019) and mildly resistant to the ascomycete *Macrophomina phaseolina*, which is responsible for charcoal rot disease in melons (de Sousa Linhares et al. 2020).

**Fig. 2:**
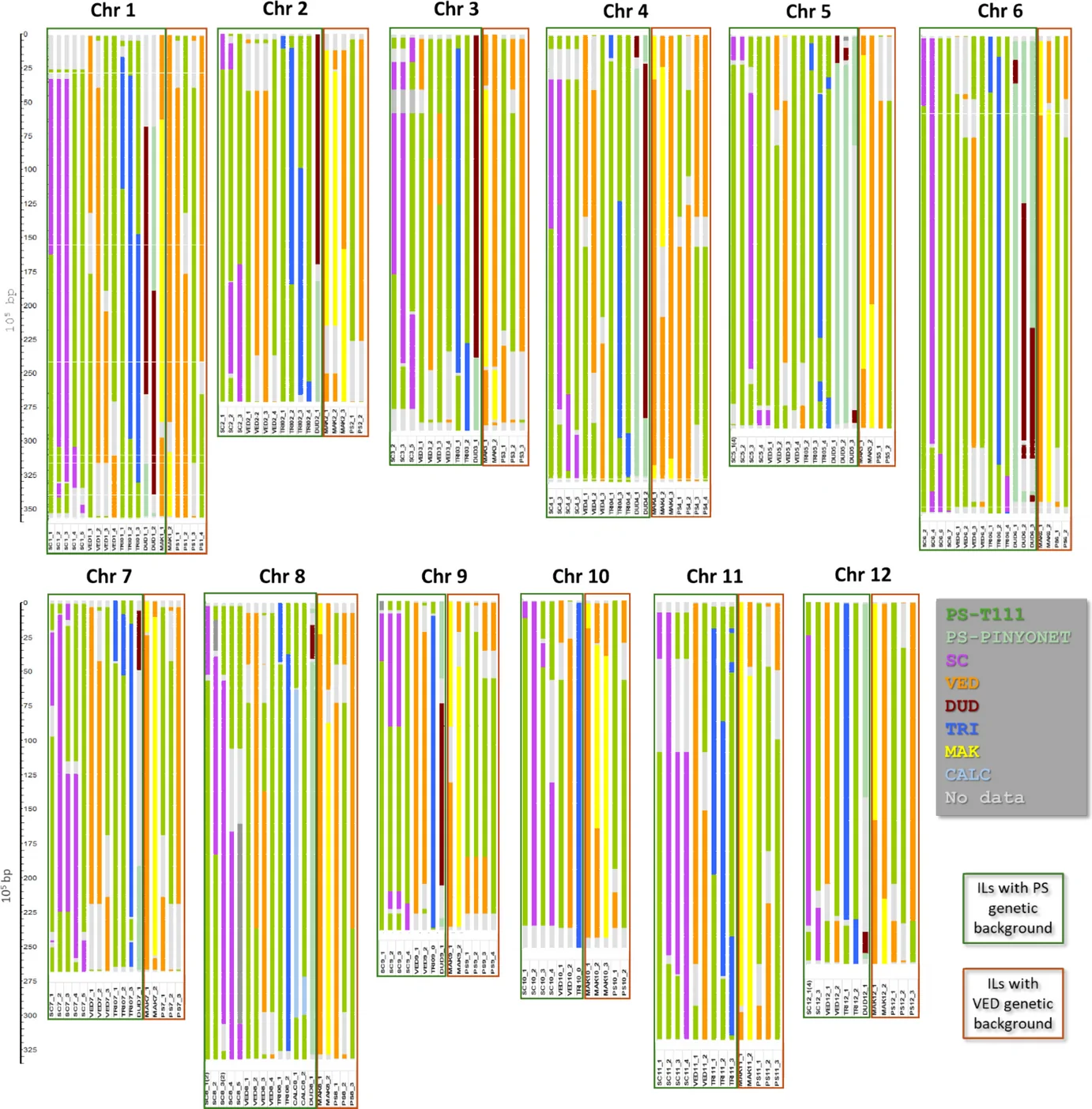
Graphical genotypes of the melon introgression line (IL) libraries based on genome‑anchored markers distributed along the 12 melon chromosomes (Chr1–Chr12). The physical positions (bp) are indicated on the left-hand scale. Donor-introgressed segments and recurrent genetic backgrounds are represented by different colors according to the legend on the right. Green and orange indicate the ‘Piel de Sapo’ (PS) and ‘Védrantais’ (VED) genetic backgrounds, respectively. The light grey regions indicate intervals containing recombination breakpoints for which the precise position could not be determined because no informative markers were available between adjacent donor and recurrent-parent markers. The extent of these intervals depends on marker density and genotyping platforms used in the different IL libraries. Further details are given in Supplementary File 4

In summary, these accessions displayed high variability in key fruit traits, including morphology, soluble solids content, aroma, ripening behavior, and resistance to biotic stress. Given their different origins and uses, they may potentially adapt to different growing conditions. The six IL libraries and the two ILs derived from CALC are summarized in Supplementary File 3, and their graphic genotypes are shown in Fig. 2 and Supplementary File 4. Altogether, these can be considered as a meta-IL collection.

The SC library (Eduardo et al. 2005) includes 57 ILs, covering more than 85% of the SC genome. Argyris et al. (2017), using an Illumina 768 SNP-GoldenGate platform for a subset of 47 of these ILs, reported an average of 1.3 introgressions per IL and estimated the average size of 9.8 Mb per introgression. More recently, another IL library with a PS-T111 background was constructed employing VED as a donor (Santo Domingo et al. 2022a) in parallel to its reciprocal PS-T111 IL library in the VED background (Pereira et al. 2021, Supplementary File 2). The donor genome representation was estimated to be nearly 100% in both reciprocal IL libraries (Supplementary File 3). The PS-T111 and VED libraries included 34 and 36 ILs, respectively, with 1.1 and 1.4 introgressions per IL, respectively. The average introgression size was 18.2 and 14.6 Mb for the PS-T111 and VED IL libraries, respectively. Two other IL libraries employing PS GBs have been developed: the DUD IL library in the PS-Pinyonet background (Castro et al. 2019) and the TRI IL library in the PS-T111 background (Díaz et al. 2017; Campos et al. 2023). The DUD library included 16 ILs, covering 62% of the exotic donor genome, with an average of 3.2 introgressions per IL and an average introgression length of 8.4 Mb (Supplementary File 3) (Perpiñá et al. 2016). Only five ILs were genotyped in-depth using genotyping-by-sequencing (GBS). A small set of TRI ILs were initially developed by Díaz et al. (2017), the final 30-IL TRI library was completed by Campos et al. (2023) and genotyped with 2,590 SNPs using seqSNP technology. The percentage of wild TRI genome representation was estimated to be 91%, with an average of 1.4 introgressions per IL and an average size of 11.2 Mb. Twenty-two ILs were evaluated for fruit traits using three different assays. Two additional ILs (CALC8-1 and CALC8-2) with CALC as the donor were also developed (Díaz et al. 2014; Martinez-Martinez et al. 2022, Supplementary File 3, Fig. 2).

With respect to the libraries in the VED background, apart from the PS-T111 IL library reciprocal to the VED IL library, Perpiñá et al. (2016) developed a library of 27 ILs from MAK (Supplementary File 3). This library covers 90% of the MAK genome, with an average of 10.9 Mb per introgression and 2.7 introgressions per IL (Perpiñá et al. 2016).

## Phenotypic diversity and characterization of il libraries

Melon IL libraries have been studied extensively, especially for fruit traits, but also for other characteristics, such as disease resistance, seed size, and root morphology. Therefore, this review focuses on fruit and disease resistance traits. A snapshot of the external fruit phenotypic diversity among ILs within each library can be found in Supplementary File 5.

### Fruit morphology

Wild melons produce small (30–50 g), oval-shaped fruits without edible flesh (Díaz et al. 2017; Pitrat 2017). After domestication, ancient farmers selected for increased fruit mesocarp edible flesh, fruit size, and diverse fruit shapes (Monforte et al. 2014). Present-day melon cultivars (including landraces and traditional and modern cultivars) exhibit impressive fruit morphological diversity. Fruit sizes range from small (100–400 g), medium (400 g to 1 kg), large (1 to 5 kg), and very large (up to 35 kg), with shapes varying from flat to highly elongated. Cultivar groups and market classes have specific fruit morphologies that define each group and class (Pitrat 2017). Therefore, cultivar groups from India to Far East Asia include acidulus (small, oval, or elliptic), momordica (small to medium, flat to elongated), and makuwa (medium, round, oval, or pear-shaped). Flexuosus melons (cultivated from Mediterranean countries to India) are notably elongated and occasionally very large. Other cultivar groups cultivated from Turkey to India include dudaim (round, ovoid, and small), chandalak (round, flat, and medium-sized), indicus (elliptical and medium-sized), ameri (oval, cylindrical, and medium-sized), and cassaba (pear-shaped and medium-to large-sized). Finally, melon groups originating in the Mediterranean area include ibericus (elliptical, medium-to large-sized), inodorus (round, medium-sized), and cantalupensis (flat, round, oval, medium-sized).

The genetic basis of melon fruit morphology has been extensively investigated. Périn et al. (2002b) published the first genome-wide QTL mapping report, followed by several other studies. Monforte et al. (2014) and Pan et al. (2020) reviewed these studies and defined five and 33 consensus QTLs, respectively, based on their detection in multiple mapping populations, respectively. SC, CALC, and MAK ILs were included in at least one of these previous reports, whereas VED, PS, DUD, and TRI ILs could not be included because they were released later.

Focusing on the current ILs, 56 ILs showed effects on FSI (fruit shape index calculated as length-to-width ratio; FL/FD), inducing elongated (23) or round (33) fruits, confirming the complex genetic control of this trait within melon germplasm. The effects were confirmed in at least two independent trials for 28 ILs (50%, Supplementary File 6 A). Assuming that ILs from different libraries carrying overlapping introgressions correspond to the same underlying locus, the minimum number of QTLs involved in FSI across the IL libraries was estimated to be 20 (Table 1). Within each IL library, allelic effects with opposite directions were observed, with 14 ILs exhibiting effects on FSI that were opposite to those expected from the donor parent phenotype (i.e., transgressive effects; Table 2). Furthermore, ILs harboring similar introgressions on chromosomes 1, 5, 7, 8, 11, and 12 showed contrasting directions of FSI effects among libraries (Table 3). In IL libraries sharing the same genetic background (e.g. SC, TRI and VED in PS-T111, or MAK and PS in VED), these contrasting effects are most likely explained by allelic differentiation among donor genotypes. In contrast, comparisons among IL libraries developed in different genetic backgrounds do not allow discrimination between allelic differentiation and genotype × genetic background interactions (G × GB), because both effects are confounded.

**Table 1:** Number of introgression lines (IL) with effects on the most commonly studied traits. The minimum number of QTLs defined by these ILs and the causal genes identified are also indicated: (1) Martinez-Martinez et al. (2022), (2) Rios et al. (2017), (3) Santo Domingo et al. (2024), and (4) Guiu-Aragonés et al. (2015)

Trait	ILs	Minimum QTL number	Cloned gene
Fruit Shape Index (FSI)	28	20	*CmOFP13*(1)
Fruit Weight (FW)	36	14	
Fruit Flesh Proportion (FFP)	5	5	
Ripening related traits	24	10	
Ethylene production	5	4	*CmNAC-NOR* (2), *CmERF024* (3)
Presence/absence of fruit aroma	8	9	
Fruit firmness (FF)	23	11	
Sugar accumulation traits	30	12	
External fruit color	17	8	
Flesh color	10	5	
Fruit sutures	4	1	
Fruit netting	11	9	
Cucumber Mosaic Virus resistance	1	1	*CmVPS41* (4)

**Table 2:** Introgression lines showing transgressive phenotypes compared with introgression donor genotype. The possible candidate genes for fruit morphology according Pan et al. (2020) are also indicated

Trait	Transgressive effect	ILs	Candidate genes
Fruit Weight (FW)/Fruit Area (FA)	Increase	MAK_2-1	*CmCSR1*
		TRI05-2, VED5.1, MAK_5-1	*CmCSR2*
		SC6-4, VED6.4	*CmCNR1*
		SC11-2, MAK_11-1, MAK_11-2	*CmCNR9, CmCYP78A8*
	Decrease	PS4.1	
		PS10.2	*CmCNR6*
Fruit shape Index (FSI)	Elongated shape	SC1-4	*CmOFP12-16a, CmOFP14, CmSUN7-8*
		TRI02-1, TRI02-2	*CmACS7, CmSUN25-26-27a*
		TRI03-2	*CmSUN23b*, *CmOFP10*
		VED5.1	*CmYAB2, CmTRM5*, *CMSUN19a*
		TRI06-1	*CmSUN17-18b, SmSUN19b, CmOFP12-16b*
		MAK_7-2	*CmSUN6, CmOVATE, CmSUN13-14b*
		TRI10-0	*CmOFP13b*
		MAK_11-2	*CmTRM6-7-8b, CmSUN17-18a, CmSUN13-14a*
		TRI12-1	*CmOFP6-19c*
	Round shape	SC4-4, PS4.4	*CmSUN12, CmOFP6-19b, CmOFP13a, CmOFP1b*
		SC6-4	*CmSUN17-18b, SmSUN19b, CmOFP12-16b, CmTRM25a, CMSUN21a, CMTRM21*
		CALC8-1	*CmOFP13*
		SC12-1	*p(CmCLV3)*
Fruit Flesh Proportion (FFP)	Increase	TRI05-2	
Soluble Solid Concentration (SSC)	Higher SSC	SC3-5	
		VED7.1, TRI07-2	
		TRI10-0, MAK_10-1	
Ripening	Increase climacteric response	SC3-5	
		SC6-3	
	Slow down climacteric response	SC10-2	
Fruit Firmness (FF)	Increase FF	SC2-2, VED2.2, VED2.3	
		TRI03-2	
		SC7-2	
		SC10-2, MAK_10-1	
		VED11.2	
		TRI12-1	
	Decrease FF	PS2.1

**Table 3:** Introgression lines (ILs) sharing the same genomic region among libraries but with opposite allelic effects

Trait	Increase	Decrease
Fruit weight (FW)	MAK_2-1	DUD_2-1
	SC6-4, PS6.1	TRI06-2, MAK_6-2
	PS7.1	VED7.1, VED7.2, TRI07-2
	MAK_11-2	TRI11-2
Fruit Shape Index (FSI)	SC1-4	DUD_1-2
	TRI02-1, TRI02-2	DUD_2-1
	VED5.1	DUD_5-1
	TRI07-2	MAK_7-2
	SC8-2	VED8.2
	SC8-3	CALC8-1
	MAK_11-2	VED11.2
	TRI12-1	SC12-1
Soluble Solid Concentration (SSC)	SC3-5	MAK_3-2
	TRI07-2, VED7.1	SC7-1, SC7-2, SC7-3, SC7-4
	PS8.2	SC8-4
	TRI10-0, MAK_10-1	VED10.2
Ripening	VED6.3, VED8.1	PS6.2, PS8.2
Fruit Firmness (FF)	VED2.2, VED2.3	PS2.1, SC2-2
	TRI03-2	SC3-5
	PS8.2	VED8.2, SC8-2
Fruit sutures/ribs	PS11.1, PS11.3	VED11.1, VED11.2

The only monoecious IL (TRI02-2) exhibited elongated fruits, likely as a consequence of pleiotropic effects associated with the wild allele at the monoecy locus on the fruit shape. Similarly, the only IL-producing fruit with five pentamers (SC12-1) displayed round fruits, consistent with previous reports indicating that an increased number of pentamers is associated with a round fruit shape (Périn et al. 2002b).

ILs displayed important differences in effect magnitude, with 20 ILs showing large effects (e.g., VED11.2, PS4.4, DUD_4-2, TRI02-2, CALC8-2, MAK_7-2, SC6-4, and SC8-3). Thus, the manipulation of a small number of QTLs is likely sufficient to modify FSI. Notably, introgressions in the same genomic regions on chromosomes 6 and 8 affected FSI in at least three populations and may reflect QTLs with important roles in cultivar diversification.

Among the 35 consensus FSI QTLs defined by Pan et al. (2020), plus two additional QTLs reported by Oren et al. (2020), 32 were represented in at least one IL displaying effects on FSI (Supplementary File 6 A). Additionally, a few QTLs on chromosomes 3, 5, 9, and 11 were not represented among the highlighted ILs. Therefore, current IL libraries capture a large proportion of the genetic variability for FSI within melon germplasm. Furthermore, the recurrent presence of *SUN* and *OVATE* family members within FSI-associated introgressions (Table 2) is consistent with their well-established role in fruit shape regulation and further supports their role in melon FSI variation.

Fifty-five ILs significantly affected fruit weight (FW), and 41 fruit area (FA). In most cases, ILs reduced FW/FA (39 ILs), as the donor parents typically produced smaller fruits than the recipient parents. Twenty-nine ILs (52.73%) displayed the effects of FW in at least two different trials, and a total of 36 ILs were highlighted (Table 1, Supplementary Files 6 A and 7). The minimum number of QTLs was 14. Between four (DUD, PS) and 12 (TRI) highlighted ILs per library modified FW. The large number of ILs with reduced weight observed in TRI-ILs reflects the small fruit size of TRI, a wild accession, and suggests that the increase in FW after melon domestication involved multiple loci (potentially involving numerous genes). Additionally, 15 ILs showed transgressive effects, with 13 increasing FW and two decreasing it (Table 2). The ILs MAK_2-1, TRI05-2, SC6-4, MAK_11-1, and MAK_11-2 consistently increased FW in several trials. The most striking was TRI05-2, which included a wild TRI introgression. Differences in effect magnitude were also observed, and it was common to find ILs with a high effect on FW (for example, VED7.1, PS7.1, DUD_2-1, TRI04-3, TRI08-2, MAK_6-2, SC4-3, and SC6-4). Introgressions on chromosomes 2, 4, 5, 6, and 8 induced variation in FW in at least three libraries. Introgressions on chromosomes 2, 6, 7, and 11 showed contrasting effects on FW among the IL libraries (Table 3).

Every genomic region previously associated with FW (Pan et al. 2020; Oren et al. 2020; Cai et al. 2024; Lian et al. 2021, Supplementary File 6 A and 7) showed also an effect on FW in at least one IL and, also, only two FW QTLs on chromosomes 5 and 9 are not represented among the highlighted ILs (Supplementary Files 6 A and 7). Furthermore, DUD_1-2, PS4.1, TRI07-2, and PS7.1 showed effects on FW, but the introgressed genomic regions have not been associated with FW in previous studies. Thus, the IL libraries captured a high level of allelic diversity for fruit weight (FW) within melon germplasm and recapitulated nearly all major FW QTLs reported in previous studies.

Overall, the limited overlap between introgressions affecting FSI and FW suggests that these traits are largely controlled by distinct genetic factors. Similar to the pattern observed for FSI, members of the CNR and CRS gene families are frequently found within FW-associated introgressions (Table 2), supporting their role as candidate genes underlying FW variation.

Fruit flesh proportion (FFP) was studied in four IL libraries (DUD, MAK, SC, and TRI), although no replicated trials were performed for the SC library. Thirty-two ILs showed effects on FFP, with four increasing and 28 decreasing, but only five showed consistent effects in replicated trials (DUD_1-2, MAK_2-2, TRI05-2, TRI06-2, TRI08-2, Table 1). The introgression on TRI08-2 overlapped with a genomic region with a significant selective sweep between wild and cultivated agrestis (Zhao et al. 2019), suggesting its possible role in increasing FW and FFP during domestication. TRI05-2 showed the most striking phenotype, consistently increasing FFP, even though the introgression belonged to a wild melon (Table 2).

#### Advanced studies on fruit morphology

Fernandez-Silva et al. (2010) studied the Genetic-x-GB (GxGB) interactions of FSI QTLs from SC4-4, SC6-4, and SC12-1 introgressions with an array of modern melon inbreds. They found that these introgressions generally had consistent effects across different GBs. Similarly, Perpiñá et al. (2025) found consistent effects across Spanish traditional variety backgrounds for the ILs TRI08-2 and TRI05-2 for FW and CALC8-1 and DUD_4-2 for FSI, whereas differences in magnitude effects across GBs were observed for VED11.2 and DUD_4-2 for FW. In addition, Martinez-Martinez et al. (2022) observed that the effects of *fsqs8.1* included in CALC8-1 were evident across a wider range of GB, although a significant G × GB interaction was observed. Specifically, *fsqs8.1* had a greater effect on cultivars with elongated fruits than on those with round fruits.

The first study on epistatic interactions for FSI among introgressions was conducted by Fernandez-Silva et al. (2009) to explain the heterosis for FSI observed in the hybrid SC × PS, which exhibited extremely elongated fruits compared with both PS and SC. Pairwise combinations between SC6-4, SC12-1 (round fruit), SC8-3, and SC10-2 (elongated fruit) revealed the absence of epistatic interactions, except for a modest negative epistatic interaction between SC8-3 and SC12-1. Elongation alleles showed dominant gene action, whereas round-fruit alleles were additive or recessive. The authors concluded that the observed heterosis was mainly attributable to dominance complementation.

Riahi et al. (2020) also found epistatic interactions among TRI introgressions on chromosomes 6 and 11 in FFP. The combination of both introgressions reduced FFP, whereas no significant effects were found for single introgression. In further studies, Campos et al. (2023) found that IL TRI06-2 (lacking the chromosome 11 introgression) showed a significant reduction in FFP. The detection of QTL effects on chromosome 6 may depend on the statistical power of the experimental design and/or environmental effects, which could explain the inconsistencies observed across studies. Nevertheless, the combination of both introgressions had a strong effect on FFP, indicating the possible role of epistatic interactions during melon domestication.

Díaz et al. (2014) also found epistatic interactions between the gene *a* (controlling female flower sex expression) and the QTL *fsqs8.1* in a cross between PS and PI 124112: CALC *fsqs8.1* allele suppressed the pleiotropic effects *a* on fruit elongation. Martinez-Martinez et al. (2022) introduced *fsqs8.1* from PI 124112 in the monoecious cultivar ‘Mucha Nesvi’, generating a monoecious breeding line with round fruits, demonstrating the usefulness of this QTL for modifying fruit morphology in monoecious GBs.

Fernandez-Silva et al. (2010) conducted the first fine-mapping study focusing on SC4-4 (FW), SC6-4, and SC12-1 (FSI). However, the mapping resolution achieved for SC4-4 and SC6-4 was insufficient to identify candidate genes, largely due to the unavailability of the melon reference genome at that time. Regarding SC12-1, the QTL was mapped to a 5 cM interval and co-segregated with the carpel number gene (*p*), supporting that the variation of FSI was due to a pleiotropic effect of carpel number variation. Wang et al. (2022) identified the gene underlying this variation in carpel number (*CmCLV3*), which is a strong candidate for the round-fruit phenotype observed in SC12-1.

Martinez-Martinez et al. (2022), using a map-based cloning approach, demonstrated that the gene underlying the FSI QTL *fsqs8.1*, introgressed in the PS-T111 background from the Indian accession PI 124112 in the IL CALC8-1, corresponds to *CmOFP13,* a member of the Ovate Family Protein (OFP) gene family*.* The induction of a round shape was associated with increased expression of *CmOFP13* during early ovary development in CALC8-1, before the formation of the stigma, style, and anthers with pollen. Comparison of the coding sequences of PS-T111 and PI 124112 revealed no non-synonymous mutations. In contrast, structural variations, including deletions of several kbs both upstream and downstream of *CmOFP13,* were identified in the CALC8-1 genome. These structural variations likely affect regulatory regions and may account for the differential expression of *CmOFP13* observed in CALC8-1. Mayobre et al. (2025) developed a loss-of-function CRISPR-*Cas9* mutant of *CmOFP13* in the VED background. Mutant VED plants produced more elongated ovaries and fruits, further demonstrating the role of *CmOFP13* in organ morphology. *CmOFP13* is likely a homologue of *SlOFP20,* a member of the tomato OFP gene family, which is different from the originally identified *OVATE* gene (Liu et al. 2002). Wu et al. (2018) found that *SlOFP20* also affects tomato fruit morphology, interacting with *TRM5* a member of TONNEUAU1 RECRUIT MOTIVE gene family. Homologues of *SlOFP20* and *TRM5* are also affecting fruit morphology in other species as *A. thaliana* (Hackbusch et al. 2005), cucumber (Colle et al. 2017; Wu et al. 2018), watermelon (Pan et al. 2020), peach (Zhou et al. 2020), pepper (Borovsky et al. 2022) and tuber shape in potato (Wu et al. 2018). Therefore, the interplay between *SlOFP20* and *TRM5* is a common genetic mechanism that underlies the morphology of fruits and other organs in diverse species. Notably, despite carrying the round allele of *CmOFP13*, PI 124112 produced elongated fruits, indicating the presence of a modifier that suppresses the effects of *CmOFP13*. Moreover, *CmOFP13* consistently exhibits effects across diverse melon GBs (Martinez-Martinez et al. 2022), similar to the dependency of *SlOFP20* effects on the GB and allelic combinations with TRM5 in tomato. These findings underscore the complexity of genetic interactions that shape fruit morphology and highlight the importance of understanding the genetic modifiers influencing the expression of key genes, such as *CmOFP13,* in melon domestication and breeding efforts.

### Fruit quality

Fruit quality is a combination of several attributes that ultimately determines the degree of consumer acceptance. The main components of fruit quality are color, texture, sweetness, morphology, flavor, and ripening, and different IL collections have been used to phenotype several traits related to these components, which are described below.

#### Fruit ripening

Ripening is the final step of fruit development, affecting fruit quality and shelf life. Fruits are classified as climacteric or non-climacteric depending on the presence of a peak in ethylene production associated with increased respiration during ripening. Ethylene is the major hormone regulating climacteric fruit ripening, and its production is considered an indicator of ripening initiation (Pech et al. 2008). In melon, however, ripening behavior appears to span a continuum rather than fitting a strict binary classification. Obando-Ulloa et al. (2008) and Vegas et al. (2013) compared representative accessions with contrasting ripening behavior and reported high ethylene production in the climacteric VED, lower but detectable ethylene production in SC, and virtually no ethylene production in the non-climacteric accession PS-T111. Although no systematic survey of ethylene production has been conducted across the diversity of melon germplasm, these results support the existence of substantial variation in the intensity of climacteric ripening.

Other ethylene-dependent indicators of fruit ripening include abscission, external color change, firmness loss, and aroma production, whereas ethylene-independent ripening traits mainly involve sugar and carotenoid accumulation in the flesh. Consistent with the variation observed for ethylene production, melon horticultural groups differ markedly in these ripening-associated characteristics (Pitrat 2017). Non-climacteric groups, such as ibericus, cassaba, acidulus, and kachri, are typically non-abscising, weakly aromatic, late maturing, and characterized by extended shelf life. At the opposite end of the spectrum, cantalupensis and makuwa accessions commonly exhibit fruit abscission, enhanced aroma production, and reduced shelf life. Intermediate combinations of these traits are observed in groups such as chinensis, ameri, indicus, and chandalak. Additional groups, including momordica and dudaim, display distinctive ripening traits, further illustrating the continuous and complex nature of ripening variation in melon.

IL libraries have been also used to map QTLs related to fruit ripening and, in some instances, the identification of the underlying genes (Pujol and Garcia-Mas 2023). Over 24 ILs have shown effects on different fruit ripening-related traits, delimiting at least 10 QTLs, highlighting the complex genetic control in melon (Supplementary File 6B). Five ILs (SC3-5, SC6-4, SC10-2, PS8.2, and VED8.2) modified ethylene production during fruit development or postharvest, defining at least four QTLs (Table 1). PS8.2 and VED8.2 showed opposite effects on ethylene production, with PS8.2 decreasing and VED8.2 increasing (Table 3).

SC3-5 and SC3-5–1 induced climacteric ripening in the SC IL library (Moreno et al. 2008; Vegas et al. 2013), defining the QTLs *ETHQB3.5* and *ETHQV6.3*. When combining *ETHQB3.5* and *ETHQV6.3*, an epistatic effect was observed, accelerating the climacteric response when the alleles of SC for both QTLs were introgressed in the PS-T111 GB. The gene underlying *ETHQV6.3*, the NAC transcription factor *CmNAC-NOR,* was characterized and confirmed to be a key regulator of ripening in melon and other species (Rios et al. 2017). More recently, a knock-down mutant of *CmNAC-NOR* was obtained by CRISPR/Cas9 gene editing, which showed the absence of ethylene and aroma production and resembled the phenotype of a non-climacteric melon type (Liu et al. 2022). As for *ETHQB3.5*, recombinant ILs containing overlapping introgressions from SC were obtained, which allowed the reduction of the genomic interval containing the QTL; however, the gene underlying *ETHQB3.5* remains unknown (Dos-Santos et al. 2023). The fruit from DUD_3-1 and DUD_6-2 also showed abscission layer in genetic intervals, the introgression of these ILs harbor the genomic region that contains *ETHQB3.5* and *ETHQV6.3* (Castro et al. 2019), being probably allelic variants of those QTLs. Abscission was also observed in TRI08-1 and TRI08-2 (Campos et al. 2023), which share genomic regions with VED8.1, VED8.2, and VED8.3, where QTLs associated with climacteric ripening have been identified (Mayobre et al. 2024b).

MAK_7-2 and MAK_10-1 from the MAK library displayed delayed ripening (Perpiñá et al. 2016). Notably, MAK_10-1 was characterized by delayed flesh softening and pigmentation, enhanced sugar accumulation, and inhibition of abscission layer formation and volatile production in the whole fruit (Perpiñá et al. 2017). As MAK_7-2 also harbors an additional introgression on chromosome 10 that overlaps with MAK_10-1, so the QTL on chromosome 7 needs to be confirmed.

The two reciprocal IL collections between VED and PS-T111 (Pereira et al. 2021; Santo Domingo et al. 2022a) allowed the validation of QTLs for climacteric ripening previously described in mapping populations, as *ETHQV6.3* (*CmNAC-NOR,* Vegas et al. 2013) in PS6.2 and VED6.3 and *ETHQV8.1* (Pereira et al. 2020) in PS8.2 and VED8.2. The gene underlying *ETHQV8.1* was recently identified as the *ethylene-responsive transcription factor ERF024* (*CmERF024*) (Santo Domingo et al. 2024). All ILs in the VED GB containing PS-T111 introgressions were climacteric, although a few presented delayed ripening and/or a less intense climacteric phenotype than the climacteric parent (Pereira et al. 2021). Ripening QTLs in this IL collection co-localized with *CmNAC-NOR* and *CmERF024*, and additional QTLs for chlorophyll degradation, aroma production, and earliness of ripening were detected in PS2-2 and PS11-3. ILs in the PS-T111 background with introgressions from VED were mostly non-climacteric, with a few lines displaying mild climacteric symptoms (Santo Domingo et al. 2022b), also co-localizing with *CmNAC-NOR* and *CmERF024*, confirming that these two transcription factors are major regulators of fruit ripening in melon.

Studies of CmNAC-NOR and CmERF024 have provided valuable insights into the molecular mechanisms underlying climacteric fruit ripening in melon. *CmERF024* shows a peak of expression a few days before the onset of ethylene production and *CmNAC-NOR* expression, suggesting that it may be participating in the transition from fruit growth to fruit ripening. *CmERF024* does not regulate the expression of ethylene biosynthetic genes but rather affects epigenetic modifications (Santo Domingo et al. 2024). *CmERF024* CRISPR/Cas9 mutants exhibited suppression of ethylene production and a significant delay and attenuation of fruit ripening (Santo Domingo et al. 2024). RNA-seq analysis of the PS-T111 and VED lines demonstrated a significant transcriptomic shift beginning at 30 DAP in VED during climacteric ripening, coinciding with the peak of expression of *CmERF024*, which was not observed in the non-climacteric PS-T111 (Santo Domingo et al. 2024). In contrast, *CmNAC-NOR* may act similarly to its orthologues in other climacteric species (Rios et al. 2017).

Recently, QTL pyramiding was performed using ILs carrying the climacteric alleles (VED) of *ETHQB3.5*, *ETHQV6.3*, and *ETHQV8.1* in the non-climacteric PS-T111 background (Santo Domingo et al. 2022a), as well as reciprocal introgressions in the climacteric VED background (Estornell et al. 2026). In the non-climacteric background all three ILs individually (SC3-5, SC6-4, and VED8.2) produced a mild climacteric response in the PS-T111 GB, whereas the double and triple combinations of these QTLs in homozygosis produced melons with significant degrees of climacteric response (Santo Domingo et al. 2022a). However, when the non-climacteric PS-T111 alleles were introgressed into the climacteric VED background, significant QTL x QTL interactions affecting ripening-related traits were observed. Specifically, *ETHQV6.3* and *ETHQV8.1* acted synergistically to delay the onset of ripening, whereas *ETHQB3.5* showed an antagonistic interaction with *ETHQV6.3*, partially compensating their effects on ripening (Estornell et al. 2026). A similar phenotype was reported in MAK_10-1, carrying a MAK introgression in VED (Perpiñá et al. 2016, 2017), indicating that non-climacteric alleles of ripening QTLs modulate the climacteric response and increase fruit shelf life. These results are promising for their application in melon breeding programs for extended shelf-life in climacteric cultivars.

#### Fruit aroma: volatile organic compounds (VOCs)

Aroma is a key determinant of melon fruit quality and strongly influences consumer acceptance. Comprehensive analyses of melon germplasm have shown that aroma diversity broadly reflects both horticultural classification and ripening behavior (Obando-Ulloa et al. 2008; Esteras et al. 2018, 2020; Mayobre et al. 2024a). Based on their volatile profiles, melon accessions can be broadly grouped into aromatic and non-aromatic clusters. Aromatic melons, including most cantalupensis, ameri, makuwa, and dudaim accessions, accumulate high levels of volatile organic compounds (VOCs), particularly esters, which confer the characteristic fruity and sweet aroma of climacteric melons. In contrast, most inodorus, acidulus, flexuosus, and wild agrestis accessions produce lower total volatile levels and are characterized by reduced ester content together with higher concentrations of fatty acid-derived aldehydes and alcohols, resulting in fresh green or cucumber-like aroma notes (Gonda et al. 2016; Esteras et al. 2018). However, these categories represent the extremes of a broad continuum rather than a strict classification. Considerable variation exists within each group, and several horticultural types, including chinensis, momordica, and some ameri accessions, display intermediate volatile profiles, highlighting the complex and continuous nature of aroma variation in melon (Esteras et al. 2018, 2020; Moing et al. 2020; Mayobre et al. 2024a). The main VOCs contributing to melon aroma belong to the ester, aldehyde, alcohol, and terpenoid chemical groups. Minor compounds, such as sulfur compounds, apocarotenoids, furans, ketones, and lactones, also contribute to the overall aroma profile. The aroma of melons is closely related to ripening behavior. Climacteric varieties exhibit strong fruity and sweet aromas dominated by esters and are generally found within the aromatic group. In contrast, non-climacteric lines have a weaker aroma with fresh, green notes, driven by higher levels of aldehydes and alcohols, and are usually classified as non-aromatic (Pujol and Garcia-Mas 2023; Mayobre et al. 2024a).

As a breeding trait, the presence or absence of characteristic melon aroma has been used to detect QTLs in several IL libraries. Using this method, Perpiñá et al. (2016) identified one QTL at the beginning of chromosome 10 in MAK_10-1 that had lost the characteristic melon aroma from VED (Perpiñá et al. 2017), concomitant with the ripening delay described in the ripening section. In the DUD library (Castro et al. 2019), DUD_6-2 acquired the characteristic aroma of DUD, which was not present in PS. Climacteric ripening and aroma production were concomitantly altered in the pairs of reciprocal ILs PS6.2/VED6.3 and VED8.1, VED8.2, and VED8.3 (Pereira et al. 2021; Santo Domingo et al. 2022b, see ripening section for more details on effects on ripening). Aroma production was delayed in PS6.2 but advanced in VED6.3. Both ILs, PS6.2 and VED6.3, contained the *CmNAC-NOR* gene within the introgression on chromosome 6 (see above). The knock-down *nor-3* mutant of *CmNAC-NOR* delayed the production of aroma, whereas the knock-out *nor-1* mutant lost the aroma (Liu et al. 2022). A similar behavior was observed on chromosome 8, where three QTLs were identified for aroma production: VED8.1, VED8.2 (co-localizing with *CmERF024* (Santo Domingo et al. 2024), and VED8.3 (Santo Domingo et al. 2022b). These QTLs activated the production of aroma in PS by introgressing VED alleles. Moreover, the CRISPR/Cas9 knock-out mutant of *CmERF024* did not produce the characteristic melon aroma (Santo Domingo et al. 2024). Unexpectedly, one QTL on chromosome 11 (PS11.3) accelerated the production of aroma when the PS allele was introgressed into the VED background, as well as other ripening traits.

To improve the knowledge of melon aroma composition and its genetic regulation, solid-phase microextraction coupled with gas chromatography-mass spectrometry (GC–MS) has been used, showing high diversity and amount of volatile organic compounds (VOCs) in melon flesh and rind (Mayobre et al. 2024a). The evaluation of VOCs content has been conducted for four IL collections, SC (Obando-Ulloa et al. 2008, 2009, 2010; Dos-Santos et al. 2023), MAK (Perpiñá et al. 2021), PS and VED (Mayobre et al. 2024b).

VOCs profiles were determined in 25 SC ILs, with low variation among ILs: only SC4-3, SC4-4, and SC8-1 increased 3-Hydroxy-2,4,4-trimethylpentyl 2-methylpropanoatea and SC8-1 increased octanal (Obando-Ulloa et al. 2010). In contrast, further studies performed with SC3-5 (containing *ETHQB3.5*, see ripening section) and recombinant derived sub-ILs showed a variation in VOC content in accordance with their ripening behavior (Obando-Ulloa et al. 2008). Esters such as methyl acetate and isobutyl acetate were predominant in climacteric ILs and sub-ILs (SC3-5, 5M4, 5M9, 5M10), and aldehydes such as pentanal were predominant in the non-climacteric sub-IL (5M7). The impact of senescence on aroma was also evaluated (Obando-Ulloa et al. 2009). In senescence, non-climacteric sub-ILs reduced the aldehyde content and increased the sulfur-derived compounds, whereas in climacteric sub-ILs, the relative contribution of acetate and non-acetate esters increased, even though the relative levels of aldehydes and esters decreased. Some VOCs, such as benzyl acetate and pentanal, were proposed to be used as biomarkers at both harvest and senescence, which permit distinguishing between climacteric and non-climacteric lines. Furthermore, a mapping strategy was conducted in SC3-5 (Dos-Santos et al. 2023), and although the gene responsible for QTL *ETHQB3.5* was not identified, QTLs for individual VOCs were detected, which increased the content of acetate esters and sulfur-derived compounds in climacteric ILs.

Fifty-seven VOCs were identified in 25 ILs from the MAK library (Perpiñá et al. 2021). Interestingly, MAK decreased the quantity of VOCs, mainly alkyl esters and apocarotenoids, in comparison to VED. Based on the VOC data, two ILs (MAK_2-1 and MAK_9-2) clustered with VED, whereas the rest clustered with MAK. Some ILs exhibited interesting phenotypes. For example, MAK_1-1 produced a high amount of eugenol, a compound absent in VED, with important aromatic notes in other varieties, such as DUD, and involved in plant defense in other species, such as tomato. In the MAK_1-1 introgression, the annotated MELO3C024886 (4-coumarate:CoA ligase) was identified as a candidate gene involved in the eugenol biosynthesis pathway. MAK_4-3 showed high levels of aldehydes and apocarotenoids, with two candidate annotated genes within the introgression: MELO3C030975 (branched-chain amino acid aminotransferase) and *CmCRTISO1* (MELO3C009571, carotenoid isomerase). The influence of ripening behavior on VOCs production was observed in MAK_7-2 and MAK_10-1 ILs, which share a common region at the beginning of chromosome 10 and behave as non-climacteric, in contrast to the parental line VED (see ripening section). Ethylene is involved in the induction of some enzymes of VOCs biosynthesis, such as alcohol dehydrogenases (ADHs) and alcohol acyltransferases (AATs), and seems to regulate the expression of others, such as branched-chain amino acid aminotransferase (*CmBCAT1*) and aromatic amino acid aminotransferase (*CmArAT1*) (Mayobre et al. 2024a). The evaluation of 25 ILs at two locations demonstrated a strong environmental effect on VOCs production, as previously described (Beaulieu 2006; Bernillon et al. 2013; Zarid et al. 2020). However, some ILs were more stable, with similar VOCs profiles in both environments, including MAK_2-3, MAK_5-2, MAK_7-2, MAK_8-1, MAK_10-2, MAK_10-3 and MAK_11-2. Moreover, noteworthy QTLs effects identified in MAK_1-1 (associated with eugenol) and MAK_4-3 (associated with apocarotenoids) were also consistently detected across environments. Taken together, these findings highlight the potential of these environmentally stable QTLs for deployment in aroma-directed breeding strategies aimed at improving fruit flavor.

VOCs production was also analyzed in the two reciprocal introgression line collections between PS-T111 and VED (Mayobre et al. 2024b). In both collections, 160 VOCs were identified, and more than 1000 QTLs were detected in 51 ILs of the two populations. In general, it was observed that modifying the aroma profile of a highly aromatic melon variety, such as VED, requires altering several regions of the genome, whereas in a non-aromatic variety, such as PS, changes in small genomic regions are sufficient to affect its VOCs profile. This result was also observed in two QTL stacking studies, both when VED alleles related to climacteric ripening QTLs were introgressed into the PS background (Santo Domingo et al. 2022a, b) and when PS introgressions were combined in the VED background; (Estornell et al. 2026). In the reciprocal IL collections, some QTLs for ester and aldehyde content co-localized with QTLs linked to ripening behavior, such as those on chromosomes 3, 5, 6, 7, and 8. However, QTLs for terpenes were scattered throughout the genome. Interestingly, even though non-climacteric PS has a low quantity of esters compared to VED, some PS alleles were able to increase the ester content when introgressed in VED, such as QTLs on chromosomes 2, 6, 10, and 11. In this study, two clusters of QTLs on chromosomes 6 and 11 were fine-mapped. On chromosome 6, two enoyl-CoA isomerase2 (ECI2) genes (MELO3C006443 and MELO3C006444) were selected as putative candidate genes for the lipid-derived volatile QTL cluster. On chromosome 11, three subQTLs were identified, which showed the complex genetic regulation of the ester biosynthetic pathway.

Fine mapping of VOC-related QTLs in melon is challenging due to the large diversity of volatile compounds produced and the strong influence of environmental conditions on aroma production. To address this complexity, two RIL populations were evaluated: one derived from a cross between PS and VED (Mayobre et al. 2021) and the other between PI 414723 and Dulce (Galpaz et al. 2018). Several QTLs detected in the PS × VED RIL population were validated in the two reciprocal ILs from the same parental lines, revealing a major contribution of chromosomes 3, 5, and 8 to the regulation of the ester-aldehyde balance (Mayobre et al. 2024b). The second RIL population involved different founders. PI 414723 is an Indian non-sweet melon characterized by pale orange flesh and an unpleasant sulfurous aroma, whereas Dulce is a cantaloupe-type melon with sweet, dark orange flesh and a pleasant aroma (Galpaz et al. 2018). Both parents are climacteric, with PI 414723 displaying rapid maturation and fruit softening. In this RIL population, QTLs located on chromosomes 3, 6, 8, 10, and 11 co-localized with QTLs later described in the PS and VED IL populations. Notably, a sulfur QTL was validated in the PS6.1 region, along with QTLs associated with climacteric ripening (Galpaz et al. 2018; Mayobre et al. 2024b). The validation of these QTLs in multiple populations and across different environments indicates low environmental sensitivity and genetic stability, making them promising candidates for aroma-directed breeding.

#### Flesh firmness and postharvest quality

Textural traits are key quality parameters for market acceptance, representing an important component of consumer preference and playing a critical role in melon shelf life. A common approach to characterizing fruit texture is the measurement of fruit firmness (FF) or hardness using a penetrometer or related devices. Fruit firmness is a complex trait influenced by ripening behavior and the progressive degradation of cell wall components (Gustani et al. 2025). High FF values are typically observed in non-climacteric varieties (e.g., ibericus), whereas climacteric groups, such as cantalupensis, momordica, and makuwa, usually produce softer fruits (Bianchi et al. 2016).

In addition to firmness, other textural attributes contribute substantially to differences in fruit quality. Mealiness, a texture disorder characterized by reduced juiciness and poor tissue cohesion due to enhanced cell separation rather than cell rupture; chewiness, defined as the amount of work required to masticate the flesh to a state suitable for swallowing; and crunchiness, which reflects the perception of crisp fracture and acoustic/mechanical resistance during biting, represent complementary dimensions of texture perception. These traits are often correlated and show high variability across melon germplasm, contributing to important cultivar- and accession-specific differences (Bianchi et al. 2016; Fernández-Trujillo et al. 2008; Obando-Ulloa et al. 2008).

These textural traits display extensive variation across melon germplasm and are often associated with differences in ripening behavior and postharvest performance among horticultural groups. In general, non-climacteric groups such as ibericus and cassaba tend to retain firmness during storage, whereas climacteric groups such as cantalupensis, makuwa, and momordica are characterized by faster softening and reduced shelf life (Bianchi et al. 2016; Pitrat 2017).

In climacteric melons, the progressive loss of whole-fruit texture during ripening not only reduces sensory quality but also increases susceptibility to physiological disorders, particularly stylar-end softening and cracking, especially in climacteric fruits (Tijskens et al. 2009). Studies using SC ILs established quantitative firmness thresholds associated with increasing severity of stylar-end softening, classified as severe (< 7 N·mm⁻^1^), moderate (7–12 N·mm⁻^1^), slight (12–20 N·mm⁻^1^), and very slight (20–35 N·mm⁻^1^). Fruits with stylar end hardness values above 35 N·mm⁻^1^—typical of non-climacteric genotypes, did not exhibit cracking or over-ripening symptoms, whereas fruits with values below 20 N·mm⁻^1^ consistently developed these disorders (Martínez et al. 2009). Conversely, excessively hard fruits may also develop cracking, even under field conditions, indicating that both insufficient and excessive firmness can negatively affect fruit integrity (Fernández-Trujillo et al. 2013).

Fruit firmness has been studied in all IL libraries, although in greater detail in the SC library (Moreno et al. 2008; Dos-Santos et al. 2011; Zarid et al. 2021). Twenty-four ILs affecting FF have been reported, either increasing (15) or decreasing (9) FF, defining a minimum of 10 QTLs (Table 1, Supplementary File 6B). Transgressive ILs were frequent, with nine ILs increasing FF despite the low firmness of the donor accession (Table 2). Consistent effects of FF on chromosomes 2 and 8 were observed in three independent libraries. In addition, opposite allelic effects were detected on chromosomes 2, 3, and 8, reflecting allelic diversity at these loci (Table 3). As expected, ILs carrying ripening QTLs on chromosomes 3, 6, 8, and 10 (see ripening section) also affected FF concomitantly with their effects on ripening. Thus, climacteric ILs (e.g., SC3-5 and VED8.2) showed reduced FF, whereas the IL reducing climacteric ripening (SC10-2) showed increased FF. In contrast, ILs with introgressions on chromosomes 2, 4, 11, and 12 were not previously associated with ripening, suggesting that these loci may harbor climacteric-independent genes controlling FF. Fruit firmness has also been examined in other studies using diverse germplasm panels (Amanullah et al. 2018; Chen et al. 2023; Nimmakayala et al. 2016; Sai et al. 2022; Oren et al. 2022). Some of previously reported loci are represented among the IL libraries, except those on chromosomes 5 (Chen et al. 2023; Dai et al. 2022; Oren et al. 2022) and 9 (Nimmakayala et al. 2016), suggesting that part of the natural diversity for this trait remains unexploited.

SC 10–2 showed the higher FF among SC ILs (Moreno et al. 2008). Comparative RNA-seq analyses revealed that higher FF in SC10-2 correlated with reduced activation of ethylene-responsive cell wall disassembly genes, including polygalacturonases and expansins, and enhanced expression of genes involved in cellulose and hemicellulose organization (Zarid et al. 2021). These data indicate that firmness-related QTLs modulate coordinated transcriptional programs that affect wall architecture rather than single enzymatic steps.

Postharvest responses to cold storage have also been investigated with SC ILs. SC10-2 also displayed altered volatile organic compound profiles under cold storage conditions. Transcriptomic analyses confirmed that higher FF in SC10-2 is associated with attenuated ethylene-mediated wall disassembly and reinforcement of cellulose and hemicellulose-related pathways, supporting a model in which firmness-related QTLs regulate integrated transcriptional responses to postharvest stress.

Additionally, SC7-2, a non-climacteric firmness-enhancing IL, maintains a high FF during storage through increased contributions of tightly bound cell wall polymers, particularly cellulose- and hemicellulose-rich fractions, and reduced pectin solubilization (Moreno et al. 2008; Dos-Santos et al. 2011). However, despite its pronounced textural stability, SC7-2 displayed elevated chilling injury (CI) susceptibility under low-temperature conditions, manifested as flesh translucency and tissue collapse. Together, these results demonstrate that SC7-2 represents a firmness strategy based primarily on structural rigidity and limited wall remodeling, whereas SC10-2 exemplifies an alternative strategy combining moderated firmness loss with enhanced physiological and molecular plasticity, resulting in improved tolerance to chilling stress. These IL-based studies indicate that flesh firmness and CI tolerance are, at least in part, controlled by distinct genetic and physiological mechanisms.

Most other studies addressing chilling injury in melon have focused on commercial cultivars or diverse germplasm panels, emphasizing oxidative stress control, membrane lipid remodeling, and hormone signaling as primary determinants of CI tolerance (Sevillano et al. 2009; Ning et al. 2022; Huang et al. 2023). Although these approaches have identified conserved cold response pathways, they generally lack the resolution required to associate postharvest phenotypes with specific genomic regions. IL-based analyses uniquely bridge genetic dissection and post-harvest physiology by directly linking defined introgressions with contrasting firmness strategies and chilling responses.

From a breeding perspective, these findings indicate that selecting for high flesh firmness alone is insufficient to ensure improved postharvest performance under cold storage. Firmness-enhancing QTLs associated with rigid cell wall architectures, such as those present in SC7-2, need to be combined with loci conferring physiological plasticity and stress responsiveness, as illustrated by SC10-2. Therefore, introgression lines provide an effective framework for pyramiding complementary traits while minimizing negative effects on ripening behavior and sensory quality, enabling the development of melon cultivars with an extended shelf life and improved tolerance to chilling injury. SC10-2 appears especially promising for breeding because it combines high flesh firmness with enhanced tolerance to chilling injury, suggesting potential value for the development of melon cultivars with extended shelf life.

Other ILs of potential breeding interest include VED2.2, VED2.3, TRI03-2, PS4.1, PS11.3, PS12.2, PS12.3 and TRI12-1, which increased flesh firmness without apparent effects on other ripening-related traits (Supplementary File 6B). However, their postharvest performance and tolerance to chilling injury were not evaluated in this study. Therefore, further research will be required to determine whether these lines also provide advantages during storage and to assess their overall breeding value.

#### Fruit sweetness

Fruit sweetness is considered the main component of fruit quality in modern melon breeding. Wild melons do not produce edible flesh; therefore, flesh sugar content is considered a post-domestication trait. Ancient melon cultivars produce non-sweet fruits (Paris et al. 2012). Sweet melons likely emerged in Central Asia around the ninth century CE and reached the Iberian Peninsula by the eleventh century CE, whereas their introduction into the rest of Europe did not occur until the fifteenth century (Paris et al. 2012). In contrast, non-sweet melons are currently cultivated in Sudan (tibish cultivars, Pitrat 2017) and India (Dhillon et al. 2007, 2009, 2012). Therefore, flesh sugar content has a broad range within melon germplasm, from 2º to > 15 º Brix (Stepansky et al. 1999; Burger et al. 2002; Leida et al. 2015; Gur et al. 2017; Pitrat 2017; Tzuri et al. 2025). The variability among Mediterranean cultivars is much lower than that among Central and Far East Asian cultivars, as the cultivation of sweet melons is much more popular in the former region, with the exception of the non-sweet flexuosus and chate cultivars. On the other hand, non-sweet melons as conomon and momordica cultivars are commonly cultivated in Central and Far East regions, together with sweet melons as makuwa cultivars.

The genetic control of fruit sweetness has been investigated in more than 20 studies (see Díaz et al. 2011 and Tzuri et al. 2025 for comprehensive reviews). Tzuri et al. (2025) identified 130 sweetness-related QTLs in the literature. Approximately 85% of the melon genome has been associated with sweetness in at least one experiment, which implies a highly complex genetic control for this trait. Consequently, fruit sweetness has also been investigated among IL libraries by assessing Soluble Solid Concentration (SSC). Additionally, sucrose (SUC), glucose (GLU), and fructose (FRU) concentrations were measured in the SC, VED, and PS libraries. Forty-five ILs modified SSC; however, only 13 (29%) showed consistent effects among replicated trials. Based on the effect repeatability within or among IL libraries, thirty ILs affecting SSC were highlighted, defining at least 12 QTLs (Table 1, Supplementary File 6 C). Between one and four ILs affecting SSC were identified per IL library, except for the SC library, where 12 ILs were highlighted, probably because this library has been evaluated for SSC in a larger number of trials. Moderate to low heritability, environmental effects, and GxE interactions have been reported for SSC in several IL libraries (Eduardo et al. 2005; Perpiñá 2016; Castro et al. 2019), which might explain the low reproducibility of IL effects compared with fruit morphology traits. ILs with significant effects on SSC usually modify SSC by more than 20% compared with the recurrent parent; therefore, single QTLs with major effects on SSC would not be as frequent as those for fruit morphology traits, and QTLs with minor effects are likely to remain undetected in the IL libraries. Most ILs reduced SSC, concomitant with the low SSC of most donor genotypes. Interestingly, SC3-5, VED7.1, TR0I7-2, TRI10-0, and MAK_10-1 manifested a transgressive increment of SSC (Table 2). Introgressions on chromosomes 5 and 10 showed consistent effects on SSC in at least three libraries; therefore, these loci are candidates for involvement in the diversification of sugar accumulation across melon germplasm. Opposite allelic effects were found in introgressions on chromosomes 3, 7, 8, and 10, which might reflect allelic variation at those loci (Table 3).

A large proportion of the QTLs reviewed by Tzuri et al. (2025) were represented in at least one of the highlighted ILs, with the exception of chromosomes 1 and 9 (Supplementary File 8, Supplementary File 6C.). This finding supports the notion that the IL libraries capture a substantial portion of the allelic diversity associated with sugar accumulation. Tzuri et al. (2025) also highlighted key genomic regions on chromosomes 3, 8, and 10 through comparative analyses of multiple populations. Among the IL libraries, introgressions on chromosome 10 were associated with SSC variation in four libraries, whereas those on chromosomes 3 and 8 were detected in only two libraries. Interestingly, chromosome 5 introgressions affected SSC in all three libraries, even though chromosome 5 has not been previously identified as a major genomic region controlling this trait in other mapping populations. These differences may reflect the specific allelic variation contributed by the donor parents of each IL library, highlighting the importance of GB in QTL detection and trait expression.

A correlation between fruit size and SSC is common in tomatoes; however, weak or no correlation is usually found in melon studies (Tzuri et al. 2025). This lack of correlation was also observed among the IL libraries. For example, 12 ILs modified FW in the TRI library, but only four had effects on SSC. Similar trends can be found in the rest of libraries, except for the SC library, where seven ILs modified FW, while 12 affected SSC.

The primary sugars found in mature fruit flesh are sucrose and its hydrolysis products, glucose (GLU), and fructose (FRU). The increase in total sugar content during fruit maturation is largely attributed to the accumulation of sucrose (SUC) in the final stages of development (Burger and Schaffer; 2007; Zhang et al. 2016a). In this context, the variation in SSC, SUC, GLU, and FRU among ILs aligns with previous findings: introgressions that increased SSC were associated with elevated SUC levels (MAK_10-1, Perpiñá et al. 2017), whereas those that decreased SSC showed low SUC and/or high GLU/FRU content (for example SC5-1, SC7-4, SC8-2, Argyris et al. 2017). These results reinforce the notion that sucrose accumulation is the principal genetic determinant of sweetness in melons.

The stable consensus sugar accumulation locus, *SUCQSC5.1,* represented in SC5-1 and TRI05-1, was fine-mapped by Argyris et al. (2017) in a 56.1 kb region on chromosome 5. This region contained four predicted genes. Expression analysis in mature fruit showed that the annotated MELO3C014519, annotated as similar to BEL1-like homeodomain protein, was downregulated in SC5-1 and other low-sugar recombinant sub-ILs. Analysis of variants among PS, SC, and TRI predicted exonic variations altering interactions belonging to the homeodomain region. These findings suggest that MELO3C014519 is a strong candidate gene for sugar accumulation.

### Fruit color

Rind and flesh color are key fruit quality traits influencing consumer acceptance in melons, and both exhibit remarkable diversity across species’ germplasm. Fruit rind color (external color, ECOL) is the first visual attribute perceived by consumers and undergoes dynamic pigmentation changes throughout fruit development. Wild melons typically display a light green rind often accompanied by darker mottling, whereas cultivated melons show extensive phenotypic variation, ranging from white, yellow, orange, or green rinds to numerous shades or intensities that may appear uniform or patterned with spots and/or sutures. The final rind color results from the relative abundance and interaction of carotenoids, chlorophylls, and flavonoids (Tadmor et al. 2010). To date, three genes that determine rind color have been cloned. *CmAPRR2* (MELO3C003375), located on chromosome 4 known as *Mt-1*, regulates pigmentation likely by increasing plastid number, and controls the dark‑green and mottled versus light/white rind phenotype (Zhao et al. 2019; Oren et al. 2019; Ma et al. 2025). The second gene, *CmKFB* (MELO3C011980), located on chromosome 10, acts as a negative regulator of the flavonoid naringenin chalcone, thereby governing the development of the yellow rind (Feder et al. 2015; Gur et al. 2017). Finally, *CmKNAT2-like2* (MELO3C026289) on chromosome 2, known as *Mt-2*, has been identified to regulate mottled rind by disrupting chloroplast development and altering the expression of chlorophyll biosynthesis-related genes (Li et al. 2025).

Different effects on ECOL were observed among the IL libraries. SC3-5, TRI03-2, DUD_6-1 and PS10-1 induced yellow rind. In the case of SC3-5 and DUD_6-1, the variation of ECOL could be a pleiotropic effect of climacteric ripening in these ILs (Vegas et al. 2013; Castro et al. 2019). However, no climacteric ripening was associated with TRI03-2, even though it shares some regions of chromosome 3 with SC3-5 (Fig. 2, Campos et al. 2023). In the case of PS10-1, the yellowing could be attributed to the presence of *CmKFB* in the introgression (Pereira et al. 2021), and the effect was the opposite (dark green) for the reciprocal ILs VED10.1 and VED10.2 and for SC10-2 (Santo Domingo et al. 2022a; Eduardo et al. 2007). Light green or pale ECOL was observed in TRI04-1, TRI08-2, SC8-3, and SC8-4. The presence of an abscission layer on TRI08-2 was also reported; therefore, in this case, the variation in ECOL may also be associated with ripening behavior. However, no ripening-associated phenotype was reported for the other ILs, but the pale color in TRI04-1 could be attributed to the presence of *CmAPRR2* in the introgression. At early fruit developmental stages, VED7.1 and VED7.2 displayed a white rind, whereas PS7.1, PS7.2, SC7-2, and SC7-4 showed the opposite pattern, developing a darker green rind. These contrasting phenotypes may be linked to the *Wi* gene (Kubicki 1962), though the causal gene has not been identified yet. Finally, two reciprocal ILs, VED2.3 and PS2.2, with the absence and presence of mottled rind, respectively, could be attributed to the presence of *CmKNAT2-like2* gene*.* In summary, within the IL libraries, we detected three cloned genes controlling rind color on chromosomes 2, 4, and 10 (*CmKNAT2-like2*, *CmAPRR2,* and *CmKFB*), *Wi* gene on chromosome 7, and four additional QTLs on chromosomes 3, 6, and 8. Altogether, this accounts for eight loci influencing ECOL (Table 1), three of which are likely explained by the pleiotropic effects of ripening-related QTLs.

Melon flesh usually presents three basic colors: white, green, and orange, although variations in intensity and combinations of them result in a continuous spectrum, ranging from light-yellow or creamy to light-orange to salmon, to dark-orange and magenta, as well as from light green to dark green (Pitrat 2017). The genetic basis of flesh color is mainly controlled by two genes: *Gf* on chromosome 9 and *Wf* on chromosome 8, with a dominant epistasis of *Gf* over *Wf*. Thus, the allelic combinations *Gf* + */* + +, *gfgf/Wf* +, and *gfgf/wfwf* induced orange, white, and green flesh, respectively. The gene *CmOr*, a homologue of the cauliflower *Or* gene (Li et al. 2006), has been identified as the causal gene of *Gf* (Tzuri et al. 2015), whereas two annotated genes have been proposed as candidates for *Wf*: MELO3C003069 (Galpaz et al. 2018) and MELO3C003097.2 (Zhao et al. 2019). The founders of the IL libraries showed three basic colors, indicating different allelic combinations of *Gf* and *Wf* (Supplementary File 9). The orange flesh of VED results from the allelic combination of *GfGf/wfwf.* The white flesh of PS, MAK, DUD, and TRI is caused by the combination *gfgf/Wf Wf*. In SC, the green flesh phenotype corresponds to *the gfgf/wfwf genotype*. Consequently, all DUD and TRI IL fruits showed white flesh without segregation (*gfgf*/*WfWf*). In contrast, SC8-3, VED8.2, PS9.4, and MAK_9-2, which carry *gfgf/wfwf*, produce green-fleshed melons, whereas VED9.1, VED9.2, and MAK_8-2, which carry *GfGf/WfWf*, produce orange flesh. Notably, MAK_8-2 also displayed a yellow internal rind, differing from the typical green rind observed in most ILs and VED fruits (Supplementary File 9). This indicates that *Wf* alleles can modulate flesh color, even in a *GfGf* background. When MAK_8-2 was combined with MAK_9-2, the resulting allelic combination, *gfgf/WfWf*, generated a VED line with white flesh (Supplementary File 9). Beyond the three main flesh colors, several ILs showed variations in color intensity, such as SC7-2, SC7-4 (sharing introgressions on chromosome 7), MAK_2-3, and MAK_6-1, revealing additional QTLs independent of *Gf* and *Wf*. Altogether, five loci controlling flesh color were identified across the IL libraries (Table 1).

### Fruit external appearance

Fruit sutures (or ribs on the rind, SUT) or ribs and fruit netting (NET), two external fruit quality characteristics, have been studied in several IL libraries. While wild melons and a large proportion of melon cultivars have smooth rinds, some cantalupensis are deeply ribbed, while slightly ribbed cultivars are found within other cultivar groups, such as makuwa and chandalak. Cultivars from *c*handalak, cantalupensis, indicus, ameri, cassaba, ibericus, inodorus groups can be netted with variable extend (Pitrat 2017).

The SUT has been studied in reciprocal PS and VED libraries, where VED exhibits ribbing, whereas PS does not. All PS ILs are ribbed, except for PS11.1 and PS11.3 (Pereira et al. 2021), whereas all VED ILs are non-ribbed, except for VED11.1 and VED11.2 (Santo Domingo et al. 2022a) (Tables 1 and 3). This introgression overlaps with the genomic region previously associated with SUT (Oren et al. 2020) and includes the annotated MELO3C019694.2, which has been proposed as a candidate gene (Zhao et al. 2019). Netting is a complex trait with different inheritance patterns across GBs. SC1-3, SC2-2, SC2-3, DUD_5-1, DUD_5-2, MAK_6-2 and SC8-3 increased NET, whereas SC3-5, MAK_5-2, MAK_7-2 and SC8-4 decreased it. None of the donor genotypes (DUD, SC, and MAK) exhibited netting, whereas PS was netted. Therefore, the increased netting observed in several ILs is noteworthy, particularly in DUD_5-1 and DUD_5-2, which displayed some of the strongest and most consistent effects across replicated trials despite the absence of netting in the donor parent (Castro et al. 2019). Introgressions in SC2-2 and SC2-3 overlapped with a major NET QTL reported by Oren et al. (2020), which was later identified as *CmSN* (MELO3C010288) by Liang et al. (2023, 2024). In the current review, we highlighted nine genomic regions influencing NET (Table 1), reinforcing the complex genetic architecture underlying this trait.

### Melon ILs and virus disease resistance

Melon is affected by a large number of viruses, including Zucchini yellow mosaic virus (ZYMV), Watermelon mosaic virus (WMV), Papaya ringspot virus (PRSV), Cucumber mosaic virus (CMV), Cucurbit aphid-borne yellows virus (CABYV), Cucurbit yellow stunting disorder virus (CYSDV), Cucurbit chlorotic yellows virus, (CCYV), Tomato Leaf Curl New Delhi Virus (ToLCNDV), Melon necrotic spot virus (MNSV), Cucumber green mottle mosaic virus (CGMMV), among others (Martín-Hernández and Picó 2021). Resistance to most of these viruses is present in different accessions and varieties (Martín-Hernández and Picó 2021).

Several parental accessions used to generate the IL libraries, particularly SC and CALC, display resistance to some of the viruses described above (Table 1). However, only the SC library has been used to study resistance to CMV. Resistance to CMV in SC was previously described as recessive and oligogenic (Karchi et al. 1975), and as a quantitative trait (Dogimont et al. 2000). IL SC12‑1 showed complete resistance to several CMV strains belonging to subgroup II (Essafi et al. 2009). Following a fine‑mapping approach, the gene *cmv1*, which by itself confers resistance to subgroup II strains, was delimited to a 2.2 cM interval on chromosome 12.

The recombinant IL SC12-1–99, with shorter introgression while carrying *cmv1*, was further characterized, and it was revealed that CMV was able to replicate and move cell-to-cell in the resistant IL, but its spread was restricted to the bundle sheath (BS) cells and was never detected in the phloem cells, thereby preventing systemic infection (Guiu-Aragonés et al. 2016). This phenotype depends on the viral movement protein (Guiu-Aragonés et al. 2015). The susceptible PS-T111 allele was only needed in BS cells to allow the virus to invade the phloem and was not required to stop its replication or cell-to-cell movement.

Map-based cloning of *cmv1* identified the causal gene underlying the resistance, Vacuolar Protein Sorting 41 (VPS41) (Giner et al. 2017). VPS41 is involved in vesicle trafficking from the late Golgi to the vacuole through late endosomes, and its activity can be diverted by the virus for its translocation to the phloem. The resistant allele carried a single SNP that produced an amino acid L348R substitution, which was responsible for the resistant phenotype. Sequencing this gene in 52 melon accessions and correlating the sequences with their resistant or susceptible phenotypes to CMV-LS allowed the identification of several additional resistant accessions, which shared the same L348R mutation, while some others carried a G85E substitution. No accessions carrying both mutations were identified (Pascual et al. 2019).

Cellular localization experiments expressing *CmVPS41* identified transvacuolar strands formed only when expressing the susceptible *CmVPS41* allele, but never when expressing the allele carrying any of the mutations. Thus, the formation of transvacuolar strands correlates with susceptibility to CMV, and the mutations L348R and G85E prevent their development, stopping viral systemic infection (Real et al. 2023). SC12-1–99 has allowed to perform a comprehensive high-throughput analysis of CMV-inoculated melon plants, offering detailed insights into the proteomic changes associated to CMV infection in the susceptible (PS-T111) and resistant (SC and SC12-1–99) genotypes at different stages of infection. At the local infection stage, susceptible plants exhibited a general depletion of proteins related to translation, photosynthesis, and intracellular transport. In contrast, the resistant genotypes displayed an increase in proteins related to lipid modification and phloem function, while failing to trigger any systemic signaling event in the new leaves to prevent infection (Real et al. 2025). Finally, the resistant SC12-1–99 was used to study the behavior of *cmv1* under conditions resembling those found in nature. In the field, viruses appear mainly as mixed infections with other viruses, which cause exacerbation of symptoms due to a synergistic effect. The co-infection of SC12-1–99 with CMV-LS and two independent potyviruses allowed CMV-LS to infect the IL even when the potyvirus was inoculated using whiteflies, its natural vector, which introduced a minimal amount of virus (Giordano et al. 2023). Thus, *cmv1*-mediated resistance to CMV can be overcome when infecting in mixed infections with potyviruses, indicating that its deployment in nature would not be useful to stop CMV infections, and additional genes would be necessary to achieve resistance in the field.

Moreover, CMV strains belonging to subgroup I, such as M6 and FNY, overcome *cmv1*-mediated resistance (Essafi et al. 2009). Guiu-Aragonés et al. (2014) showed that resistance to these strains is oligogenic, involving at least three QTLs: *cmv1* on chromosome 12, with a major effect, and two additional QTLs, *qwcmv3.1* and *qwcmv10.1*, located on chromosomes 3 and 10, respectively, with an epistatic interaction between *qwcmv3.1* and *cmv1*. Combining these QTLs may also contribute to improved CMV control under mixed-infection scenarios involving potyviruses.

VPS41-mediated resistance takes place at the BS cells, preventing phloem loading. Normally, few viral particles reach the phloem cells, suggesting that the plasmodesmata (PD) in the BS cells facing the phloem could be different from other PDs (Guiu-Aragonés, 2016). The BS cells, as any other plant cell, are almost fully filled with a large vacuole, leaving the cytoplasm and organelle near the plasma membrane and, whereas in other cells the virus could go to any surrounding cell to continue the infection, in the BS cells, the virus needs to go towards the phloem. A preliminary model would imply that VPS41 protein produced by the PS allele would develop transvacuolar strands able to reach the PDs at opposite side of the BS cells allowing CMV-LS to reach the phloem, whereas the VPS41 protein encoded by the resistant allele and carrying one of the two mentioned mutations, would be unable to generate transvacuolar strands and only few viral particles, unable to develop an infection, would reach the phloem-facing PDs. As M6 or FNY strains are able to overcome VPS41-mediated resistance in the presence of the susceptible alleles of *qwcmv3.1* and *qwcmv10.1* QTLs, the model implies that the proteins encoded by them would somehow help the resistant VPS41 protein either in developing transvacuolar strands or in promoting alternative transvacuolar transport to the phloem-facing PDs.

### Other traits

Most studies using melon IL libraries have focused on fruit quality traits and disease resistance. However, ILs have also proven useful for exploring other agronomically relevant traits, although these have received considerably less attention. For example, variation in flower production was detected in several MAK ILs, with increased numbers of male flowers in MAK_5-2 and MAK_7-1 and increased numbers of female flowers in MAK_6-2, suggesting genomic regions affecting sex expression and potentially yield-related traits (Perpiñá et al. 2016). Likewise, seed weight, a trait showing extensive diversity across melon germplasm (Pitrat 2017), was affected by multiple introgressions in the SC library, with SC1-4, SC10-2 and SC12-1 producing particularly small seeds, supporting the complex polygenic control of this trait (Fita et al. 2008).

Substantial variation in root architecture was also observed among SC ILs, with SC10-2 showing a marked reduction in root length and number of root tips (Fita et al. 2008). In addition, IL populations have contributed to the genetic dissection of sulfur sensitivity, an important trait in melon production because sulfur is widely used to control powdery mildew. In agreement with previous studies, sulfur susceptibility in the SC background was associated with the introgression carried by SC1-4 on chromosome 1 (Eduardo et al. 2005), consistent with a major QTL for this trait mapped on chromosome 1 by Branham et al. (2020). Although these traits have been investigated less extensively than fruit quality or disease resistance, these examples illustrate the versatility of IL populations for studying a broad range of agronomically relevant characteristics in melon.

## Conclusions

This review highlights the successful implementation of IL libraries as powerful tools for melon genetic research. Key factors contributing to this success may include the use of common GBs, which facilitated the combination and analysis of multiple alleles across loci, and the strategic selection of donor genotypes, from wild accessions (TRI) to elite cultivars (PS, VED), capturing a wide spectrum of melon genetic diversity.

Transgressive phenotypes are frequently observed in IL populations and may arise from different genetic mechanisms. The simplest explanation is the complementation of favorable alleles contributed by both parents. However, ILs may be particularly effective for revealing transgressive variation caused by epistatic interactions because each introgression is evaluated in an otherwise uniform recipient background. The transfer of a genomic segment into a novel genetic context may modify multiple genetic interactions, generating phenotypes that extend beyond the parental range. Consistent with this hypothesis, Perpiñá et al. (2025) reported significant introgression × genetic background interactions for several melon ILs, suggesting that epistasis contributes to at least part of the transgressive variation commonly observed in these populations.

Advanced studies using melon ILs have not only identified genes underlying important agronomic traits in melon but have also contributed to a broader understanding of plant biology. While genes such as *CmNAC-NOR* confirmed the conservation of ripening regulators previously characterized in tomato, others, including *CmOFP13*, *CmERF024*, and *CmVPS41*, revealed new functions or expanded the known roles of gene families involved in fruit morphology, ripening, and virus resistance. Importantly, several of these discoveries have subsequently been validated or extended in other crop species, highlighting the value of melon IL populations as a source of biological insights with relevance beyond melon breeding.

In the short term, our research will continue to identify and functionally characterize individual genes and major‑effect QTLs controlling fruit morphology and ripening. The analysis of epistatic interactions among loci will complement those investigations, as complex traits cannot be fully explained by single‑gene effects alone. Initial studies already pointed to the existence of different types and strengths of genetic interactions underlying fruit morphology (Fernandez‑Silva et al. 2010; Riahi et al. 2020) and ripening‑related traits (Santo Domingo et al. 2022b).

Transcriptomics, metabolomics, and their interplay remain largely underexplored in melon IL libraries. The integration of IL libraries with transcriptomics, metabolomics and other multi-omics approaches represents a promising avenue for future research. A potential workflow would include: (i) identification of ILs showing contrasting phenotypes for target traits; (ii) transcriptomics and metabolomics profiling of selected ILs at relevant developmental stages or environmental conditions; (iii) integration of introgression maps with differentially expressed genes and metabolite-QTL analyses to identify candidate genes and regulatory pathways; and (iv) validation through recombinant sub-ILs, gene editing and marker-assisted breeding. Such approaches could accelerate the identification of causal genes underlying major QTLs controlling fruit ripening, aroma, sweetness, firmness and disease resistance.

Beyond trait dissection, IL libraries offer an opportunity to refine breeding objectives by linking genetic variation to consumer perception. The uniform GB of ILs allows consumer preferences to be associated with specific fruit quality traits, enabling the detection of subtle but meaningful differences, such as preferred fruit size or aroma profiles. This strategy may help to identify breeding targets that are more closely aligned with consumer preferences.

Current IL libraries were developed using Occidental cultivar backgrounds. Cultivars belonging to the conomon, momordica, and makuwa horticultural groups are appreciated in Asian countries, and active breeding programs have been developed by private companies such as Sakata Seeds (for example, Sakata Sweet cv.). The development of IL libraries in Oriental cultivar backgrounds would introduce new genetic variability in these genetic pools and help develop new cultivars adapted to consumer preferences in these regions.

Abiotic stress tolerance has been explored in melon germplasm, although much less extensively than fruit quality or disease resistance traits, and significant efforts to identify and validate tolerant accessions have emerged only recently (Saroj and Choudhary 2020; Shahwar et al. 2024; Flores-León et al. 2026). Notably, the donor accessions used to develop the current melon IL libraries were selected primarily to represent major genetic pools and horticultural diversity within the species, and recent evaluations did not reveal outstanding levels of abiotic stress tolerance among these founders (Flores-León et al. 2026). Consequently, abiotic stress has received little attention in the characterization of current melon IL libraries, likely because strongly tolerant donor sources were not available when these populations were developed. Despite the typically complex and polygenic nature of abiotic stress tolerance, studies in tomato have shown that single introgressions can confer significant levels of heat tolerance (Gonzalo et al. 2020). In the context of climate change, the identification of robust abiotic stress donors and the development of dedicated IL libraries targeting stress-adaptation traits would provide powerful tools for dissecting stress-response mechanisms and improving crop resilience.

In summary, the evidence compiled in this review demonstrates that IL libraries have become indispensable resources for dissecting the genetic architecture of complex traits in melon. Beyond enabling high‑resolution QTL mapping and gene cloning, melon ILs have refined regulatory models derived from other fleshy‑fruit species, such as tomato, while uncovering cucurbit‑specific genetic mechanisms underlying fruit morphology, ripening and adaptation. The frequent detection of strong single‑locus effects together with widespread transgressive phenotypes underscores the central role of epistatic interactions and genetic networks in shaping melon phenotypic diversity. Collectively, these advances highlight the continued relevance of developing and exploiting IL libraries as integrative platforms for fundamental research and pre‑breeding, ultimately supporting market‑oriented improvement and the long‑term adaptation of melon crops to future climatic scenarios. As such, IL libraries constitute a critical interface between basic genetics and pre‑breeding, accelerating the transfer of mechanistic insights into resilient and market‑adapted melon cultivars.

## Supplementary Information

Below is the link to the electronic supplementary material.

Supplementary file1 (XLSX 29 KB)

Supplementary file2 (XLSX 21 KB)

Supplementary file3 (XLSX 19 KB)

Supplementary file4 (XLSX 109 KB)

Supplementary file5 (PPTX 11961 KB)

Supplementary file6 (PPTX 12909 KB)

Supplementary file7 (PPTX 591 KB)

Supplementary file8 (XLSX 23 KB)

Supplementary file9 (XLSX 25 KB)

Supplementary file10 (PPTX 2646 KB)

## Data Availability

No datasets were generated or analysed during the current study.
